# Appropriately Matching Transport Care Units to Patients in Interhospital Transport Care: Implementation Study

**DOI:** 10.2196/65626

**Published:** 2024-12-13

**Authors:** Shirin Hasavari, Pouyan Esmaeilzadeh

**Affiliations:** 1 Department of Information Science & Systems Graves School of Business & Management Morgan State University Baltimore, MD United States; 2 Department of Information Systems and Business Analytics College of Business Florida International University Miami, FL United States

**Keywords:** interfacility transport care, electronic health records, data sharing, blockchain, hyperledger fabric, privacy, implementation, EMS, emergency medical services

## Abstract

**Background:**

In interfacility transport care, a critical challenge exists in accurately matching ambulance response levels to patients’ needs, often hindered by limited access to essential patient data at the time of transport requests. Existing systems cannot integrate patient data from sending hospitals’ electronic health records (EHRs) into the transfer request process, primarily due to privacy concerns, interoperability challenges, and the sensitive nature of EHR data. We introduce a distributed digital health platform, Interfacility Transport Care (ITC)–InfoChain, designed to solve this problem without compromising EHR security or data privacy.

**Objective:**

This study aimed to detail the implementation of ITC-InfoChain, a secure, blockchain-based platform designed to enhance real-time data sharing without compromising data privacy or EHR security.

**Methods:**

The ITC-InfoChain platform prototype was implemented on Amazon Web Services cloud infrastructure, using Hyperledger Fabric as a permissioned blockchain. Key elements included participant registration, identity management, and patient data collection isolated from the sending hospital’s EHR system. The client program submits encrypted patient data to a distributed ledger, accessible to the receiving facility’s critical care unit at the time of transport request and emergency medical services (EMS) teams during transport through the PatienTrack web app. Performance was evaluated through key performance indicators such as data transaction times and scalability across transaction loads.

**Results:**

The ITC-InfoChain demonstrated strong performance and scalability. Data transaction times averaged 3.1 seconds for smaller volumes (1-20 transactions) and 6.4 seconds for 100 transactions. Optimized configurations improved processing times to 1.8-1.9 seconds for 400 transactions. These results confirm the platform’s capacity to handle high transaction volumes, supporting timely, real-time data access for decision-making during transport requests and patient transfers.

**Conclusions:**

The ITC-InfoChain platform addresses the challenge of matching appropriate transport units to patient needs by ensuring data privacy, integrity, and real-time data sharing, enhancing the coordination of patient care. The platform’s success suggests potential for regional pilots and broader adoption in secure health care systems. Stakeholder resistance due to blockchain unfamiliarity and data privacy concerns remains. Funding has been sought to support a pilot program to address these challenges through targeted education and engagement.

## Introduction

### Background

Interfacility transport care (ITC) encompasses the process of transferring a patient from one acute care facility to another, with necessary clinical care provided during the transport, to avail services not offered at the sending facility [1,2]. In this context, a “facility” refers to any licensed health care entity, including hospitals, clinics, rehabilitation centers, and nursing homes. This paper specifically addresses scenarios where a patient is discharged from one facility (be it a hospital or clinic) and subsequently admitted to another as a definitive care facility. ITC practices incorporate various levels of ambulance transport services, categorized based on patient acuity. These categories include basic life support for stable patients with a low risk of deterioration, advanced life support, specialty care, and neonatal transport for patients who are stable but at medium to high risk of deterioration, as well as for those with unstable acuity [1]. Our research predominantly focuses on the latter 2 patient acuity levels, where the transporting unit must possess both the requisite infrastructure and expertise to facilitate patient transport [1,3] involved before, during, and after moving a patient from one location to another. The term “transport” refers to the physical process of moving a patient from one location to another [1]. This study examines the transfer process, beginning with the initial transport request and extending through the journey to the receiving facility.

The realm of patient transport is inherently complex, with the responsibility to coordinate the appropriate transport for each patient. However, considerable variability exists in both the individuals placing transfer orders and the information they have available [4,5]. The resolution to this issue depends on the capability of transport companies and the staff within receiving hospitals’ critical care units to effectively communicate, assimilate crucial patient information, and dispatch the most suitable transport unit accordingly. The challenge is further intensified by the wide spectrum of patient acuity and conditions, coupled with the fact that not all transport companies provide all levels of care. Thus, it is crucial to accurately match patients with the appropriate vehicle and qualified personnel [6-10]. Obstacles to this imperative match often include technological limitations, misinformation, and misunderstanding. Recognizing these challenges, high-performing transfer centers employ paramedics in dispatch centers to evaluate emergency calls and assign ambulances, thereby alleviating some of the confusion [11].

### Literature Review and Gap Analysis

In the realm of interhospital data management, 2 noteworthy blockchain prototypes, MedRec and FHIRChain, have been developed [12,13]. MedRec, using Ethereum smart contracts, is designed to organize medical records intelligently within a decentralized network. While it proposes incentives for data mining and access for research purposes, it does not address the generation of decentralized anonymous data with privacy safeguards in place. On the other hand, FHIRChain incorporates the HL7 FHIR (Health Level Seven Fast Health care Interoperability Resources) standard for clinical data and explores the possibilities of blockchain-based data sharing. The challenges posed by permissionless blockchain technology and the associated privacy concerns are acknowledged, highlighting the necessity for a permissioned blockchain approach to ensure enhanced security and user management. The discussion also includes the application of pseudonymization and encryption techniques as additional layers of data protection.

Healthchain [14] has proposed and implemented a solution based on Hyperledger Fabric (HLF) permissioned blockchain, where network members include health care providers, insurance companies, and government regulatory bodies. These entities are expected to record their data on the distributed ledger, making it accessible to all network participants. While Healthchain focuses on patients’ consent and performance metrics like network size, hardware power, batch size, and timeout, it has limitations. For instance, it uses a solo orderer—now deprecated—for network implementation, which cannot execute the consensus protocol.

Similarly, Action-EHR [15] has developed a permissioned blockchain platform designed to secure patient data within electronic health records (EHRs), either stored on the cloud or on health care providers’ premises. This platform stores only data hashes on the distributed ledger, with access to the EHRs granted solely through these hashes. Although this approach seems effective, it centralizes data and does not address issues related to single points of failure or data breaches. Furthermore, using hash functions to connect to the EHR systems and retrieve data can overload databases and slow down EHR system performance. In contrast, our proposed solution involves retrieving data from the EHR within the sending facility’s internal network at the time of the transport request, without engaging the blockchain, before directing it to the ledger.

Saeed et al [16] discuss the challenges associated with health care information sharing and explore the potential benefits of implementing permissioned blockchain technology. Another noteworthy contribution is HealthBlock [17], a proposed blockchain-based system for health care data management. HealthBlock introduces an architecture that integrates decentralized databases—specifically, it uses OrbitDB and the interplanetary file system (IPFS) to store patient EHRs. Furthermore, the system deploys a blockchain network using HLF and Hyperledger Composer. This network is designed to securely store data hashes and manage access controls during data retrieval, aiming to enhance health care data management systems’ security, privacy, and robustness while addressing their current limitations.

Currently, the primary entities facilitating data exchange (DX) are health information exchange organizations (HIOs), such as CRISP or Healthix [18,19]. These organizations enable data sharing among health care providers by connecting to their EHR systems and downloading data into their centralized storage. However, this approach presents challenges related to data governance and cost, and the centralization of data increases the risk of breaches, thereby exacerbating data security and privacy concerns. In addition, a study indicated that hospitals engaged in DX through HIOs experienced a higher incidence of IT-related breaches compared to those that did not participate [20].

Often, data are not loaded into the HIO databases at the time of patient transfer, leaving health care providers without crucial information during transport requests [21].

Many of the solutions discussed above advocate for storing entire EHRs on permissioned blockchain technology’s distributed ledgers. Alternatively, some propose saving only the hash of EHR data on the ledger while keeping the actual data on the cloud or IPFS. While we concur that permissioned blockchain technology can effectively address challenges like data security, interoperability, patient privacy, and efficient data sharing among stakeholders, it is crucial to acknowledge the health care industry’s risk-averse nature. The industry is often hesitant to embrace new technologies quickly, especially considering the significant time and financial investments involved. For instance, achieving a 96% adoption rate of EHRs in US hospitals required over US $30 billion from the Centers for Medicare and Medicaid Services since 2011 [22]. Consequently, innovative approaches should be pragmatic, not aiming to replace EHRs but rather to complement them. Our work aligns with this perspective, focusing on collecting patient data from the sending hospitals’ EHR in isolation and then disconnecting from it before involving the blockchain.

Health care is a risk-averse industry, often reluctant to adopt new technologies due to the associated time and cost implications [23]. Any innovative approach to health information sharing must acknowledge and navigate this reluctance, proposing solutions that minimize risk to existing health information systems. Institutions that maintain health care data often view this information as valuable, making them resistant to changing established practices and systems [24]. While a comprehensive blockchain–artificial intelligence solution might be a viable alternative in the future, there is an immediate need for a carefully designed, reliable approach that addresses the current challenges of EHR interoperability without compromising its security and data privacy.

Expecting the health care industry to connect its EHR systems to third-party centralized databases is a risky approach.

Currently, there is no efficient mechanism to import data from a sending hospital’s EHRs into transport requests due to privacy concerns, interoperability limitations, and the sensitivity surrounding EHR data access. These constraints lead to delays and potential risks for critically ill patients awaiting transfer. Efforts to resolve this gap are hampered by disparate systems and a lack of reliable, secure methods to share real-time data between facilities and emergency medical services (EMS) teams at the time of transport request.

### Aim and Objective

The aim of this research was to design, develop, and implement ITC-InfoChain, a secure, real-time data-sharing platform intended to seamlessly integrate critical patient data into transport requests without compromising data privacy or the security of the sending hospital’s EHRs. This platform leverages a unique interoperability solution within its architecture, designed to enhance effective dispatch and mitigate potential adverse events during patient transport. The specific objectives include assessing the platform’s technical feasibility, performance, and reliability in securely isolating, recording, and sharing patient data between facilities both at the time of transport requests and throughout the transport process. Key performance indicators include data transaction speed, reliability under varying network configurations, and overall system scalability.

## Methods

### System Architecture and Components

#### Overview

**Figure 1:**
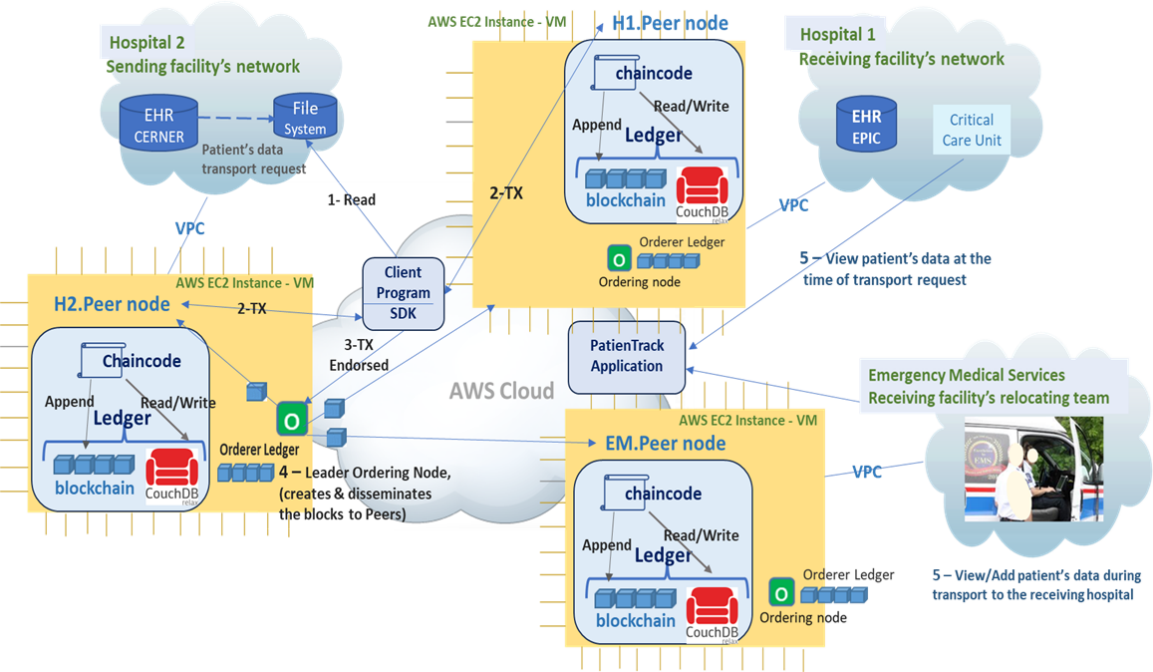
ITC-InfoChain platform architecture for real-time patient data sharing in interfacility transport (ITC) at the time of transport request and during (during) transit to the receiving hospital. AWS: Amazon Web Services; EHR: electronic health records; SDK: software development kit; TX: transaction; VPC: virtual private cloud; VM: virtual machine, EC2: elastic compute cloud 2.

Figure 1 illustrates a private cloud-based architecture of our prototype, where hospitals and EMS are involved in ITC. The hospitals are the owners of databases and ledgers on the private cloud infrastructure for security, scalability, and cost-effectiveness.

Given that the ITC-InfoChain architecture uses a permissioned blockchain to facilitate secure hospital connections, a brief background on blockchain technology provides essential context.

Blockchain technology was initially introduced in 2008 as a decentralized system for secure transactions without third-party validation, using consensus mechanisms within a peer-to-peer network. Each transaction is verified and added to a shared ledger, forming a continuous chain of blocks [25]. While often associated with cryptocurrency, blockchain has applications across various fields, including health care. Recent studies highlight its potential for secure data sharing and medical record management. In contrast to public blockchains, ITC-InfoChain uses a permissioned blockchain architecture, restricting access to authorized participants only to ensure data privacy and integrity for interfacility patient transport.

#### System Components

The ITC-InfoChain platform integrates a suite of privacy- and security-focused features designed for seamless and protected DX during interfacility transport. At the transport request stage, a dedicated DX file securely isolates patient data from the sending facility’s EHR, maintaining privacy by disconnecting after data collection. A client program then retrieves this data from the DX file, encrypts it, and connects to the permissioned blockchain network to submit transactions. Supported by the HLF network, which enables secure peer-to-peer connections among participating hospitals, ITC-InfoChain facilitates encrypted, real-time data sharing through a fully integrated and privacy-preserving design. The PatienTrack web app allows staff at the receiving facility’s critical care unit to access patient data at the time of the transport request. Staff can view any additional data entered by the transport team during the journey, ensuring continuous communication between the transport team and the receiving facility. This architecture ensures secure, scalable, and efficient data sharing during patient transport, allowing real-time updates for paramedics and the receiving hospital.

For a detailed description of the system components, client program connection, data recording process, endorsement policy enforcement, transaction ordering, consensus mechanism, transaction validation and commitment, and system crash tolerance, please refer to the Multimedia Appendix 1.

#### Key Features of Design

Key features include (1) a DX file that securely isolates patient data collection from the sending facility’s EHR at the transport request stage, by collecting data and disconnecting from it to maintain privacy; (2) a client program that reads data from the DX file and connects to the permissioned blockchain network to submit transactions; (3) an HLF network providing peer-to-peer connection among hospitals serving patient data access at the time of transport request and during transportations—it ensures strong member identity management, certification issuance, and data encryption for secure patient data sharing; (4) smart contracts for automated, compliant data sharing; and (5) PatienTrack, a web app enabling EMS and receiving hospital staff to view patient data in transport request time and during transfer.

### Key Implementation Strategy and Data Management

#### Overview

The ITC-InfoChain platform is strategically designed to ensure secure, real-time patient data sharing through an isolated DX process. At the transport request stage, the sending hospital’s EHR data are extracted and securely stored in a DX file, which remains disconnected from the EHR to uphold privacy. It reads data from the DX file and connects to the permissioned blockchain network to submit transactions to be recorded on a permissioned blockchain ledger. This setup enables real-time access for EMS and receiving hospital staff through the PatienTrack app, ensuring compliance with identity management and data encryption protocols. This strategy not only supports seamless interoperability between disparate systems but also automates data sharing through smart contracts, which enforce access control and privacy.

#### Data Management

The HLF network component of the ITC-InfoChain system adheres to privacy, security, and ownership standards by enabling hospitals to share data directly through hospital-representative nodes, without involving a third party in data management. In traditional data-sharing models, hospitals and EMS would need to send patient data to a third-party database (due to interoperability issues among EHR systems, privacy concerns with direct EHR connections, etc), which would then distribute the data to other parties. ITC-InfoChain’s peer-to-peer structure eliminates this need, allowing hospitals to retain full ownership and governance over patient data. While such traditional systems exist, they lack the capability to integrate patient data into the transport request and during transport, as they are ineffective when data needs to be instantly accessible. In addition, third-party centralized systems are vulnerable to security breaches, data privacy issues, single points of failure, and challenges with data governance [20]. This is where a permissioned blockchain framework like HLF provides a robust solution.

#### Data Lifecycle and Security

The data lifecycle involves data collection, data extraction, and recording in the distributed ledger on the HLF network, as well as real-time access at the time of transport request and during transport. Data modification is not allowed by the receiving facility’s staff and during transport, only EMS can add data to the existing ones on route to be visible to authorized members. All data are hosted on Amazon Web Services (AWS) US-based cloud infrastructure, complying with in-country data requirements.

#### Ownership, Privacy, and Compliance

Operating under HIPAA (Health Insurance Portability and Accountability Act) and health data governance policies, ITC-InfoChain ensures that data ownership remains with the originating hospital, while transparency is achieved through a shared, permissioned ledger accessible only to authorized stakeholders. Although patients do not directly access the system during transport, consent protocols are managed at the hospital level. Data are fully encrypted, with strict access controls to maintain confidentiality and data integrity across the transport process.

#### Tools Selection for the Prototype

As a peer-to-peer network directly connecting hospitals involved in ITC, we selected HLF, a permissioned blockchain technology. HLF was chosen due to its strong membership and identity management, certification issuance, and data encryption capabilities, which align with health care privacy standards. We created a subnetwork of the HLF network also known as a channel which comprised of 3 members: a sending hospital, a receiving hospital, and an EMS ITC provider. Other channels can be created in case any hospital intends to share some data mutually and confidentially or run another function on the network.

We developed the client program in Python (Python Software Foundation), chosen for its cross-platform compatibility and strong community support, which make it adaptable and easy to deploy across different systems. The PatienTrack web app was also built in Python using the Django framework. A smart contract, known as “chaincode” in HLF, was developed using the Go language, which is optimized for HLF. Go was chosen for its performance, efficient memory management, and ability to handle multiple concurrent requests. In addition, it benefits from strong community support. Other languages are also compatible for chaincode development. The Code Repository link for the client program, chaincode web app, and smart contract is provided in the checklist file in Multimedia Appendix 2 under section Item 8: Technical, Design.

### Interoperability and Data Access

#### Overview

The following workflow design and implementation thoroughly describes how the ITC-InfoChain platform interfaces with the sending facility’s EHR through the DX File, as well as how it connects with the blockchain network using HLF, software development kit (SDK) application programming interfaces (APIs), and proprietary APIs.

The ITC-InfoChain platform is designed to use a HLF connection profile, SDK APIs, proprietary APIs, and data files to facilitate interoperability as follows. We used a DX file to enable data collection and interoperability between the sending facility’s EHR and the blockchain network. The DX file collects data from the sending facility’s EHR, disconnects, and then supports subsequent data retrieval and recording on the HLF network. This setup ensures that the hospital health information system remains disconnected from the blockchain network during data extraction and recording.

When a transport request is initiated, a job setting in the sending facility’s database system triggers a connection to the EHR, collects relevant patient data, and stores it in a DX file before disconnecting from the EHR. The client program then connects to this file, automatically extracting and encrypting the data before submitting it to the peer node on the HLF network for recording. This workflow allows ITC-InfoChain to securely manage data without requiring a direct EHR connection, addressing interoperability barriers through a disconnected DX model. The client program uses a network profile to connect to the network, linking participating hospitals. In addition, a web application called PatienTrack uses SDK APIs to connect to the blockchain network to retrieve and display data to any authorized entities as members of the network.

Currently, the data shared at the time of transport request are recorded on the permissioned blockchain and are not recorded in the receiving facility’s EHR, so data standards like HL7 FHIR or SNOMED CT (Systematized Nomenclature of Medicine–Clinical Terms) have not been used. However, the platform is designed to support future integration of standards, which could enable secure, real-time, API-based data transfer directly into receiving facility EHRs. Future implementations of these standards would facilitate compatibility across a broader range of hospital systems and enhance data interoperability at national and organizational levels, enabling a more seamless data-sharing process and automated information transfer across health care systems.

#### Data Access at the Time of Transport Request Service

**Figure 2:**
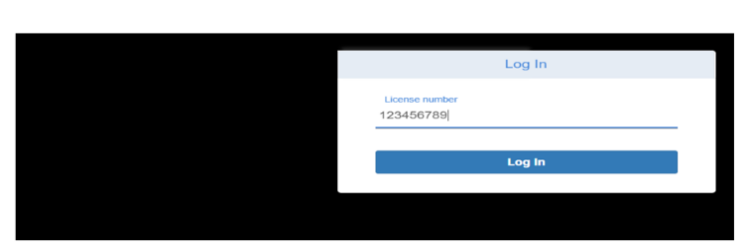
Data access at the receiving facility patient portal connecting to the PatienTrack system.

**Figure 3:**
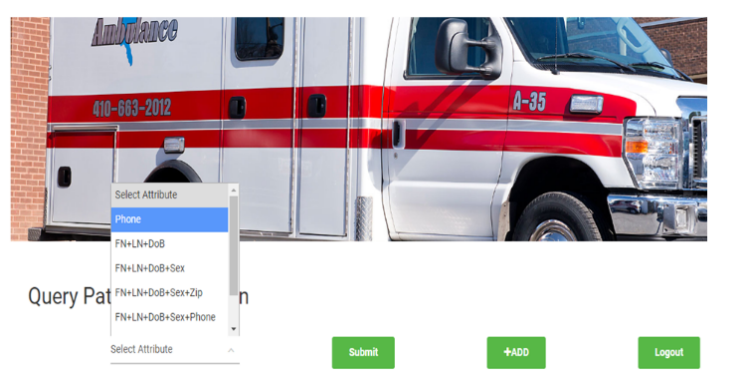
Health care providers' interface for inputting various patient identifiers to retrieve information in the PatienTrack system.

**Figure 4:**
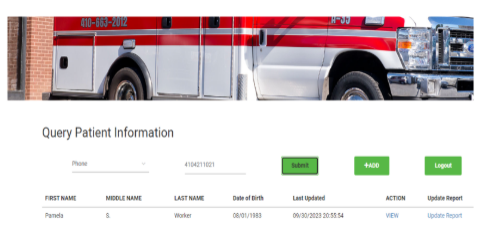
Access to patient data for critical care staff at the receiving facility at the time of transport request.

Figure 2 shows the PatienTrack web app in use during a patient transport request and transfer. Initially, a nurse from the sending hospital provides the receiving hospital’s critical care unit with basic patient information (eg, name, age, and phone number) when the hospital is out of network. Staff at the receiving hospital then enter their license number for authorization to view the patient’s critical data on a secure portal, which retrieves information from PatienTrack through the HLF network (Figures 3-4).

After reviewing patient data, staff in the critical care unit of the receiving facility can decide whether to dispatch their own transport team or call an external EMS team for the patient awaiting transport. If an EMS team is designated, the transport team can access the patient information to equip themselves with appropriate equipment and medication. They can also view the data during transport to add real-time updates on the patient’s condition, which are immediately visible to the receiving facility. This allows the facility to prepare for any necessary interventions, such as surgical rooms or specialized equipment, if the patient’s condition worsens.

Please refer to Multimedia Appendix 1 for detailed information on how patient data are accessed at the time of a transport request, including the process that allows EMS to add any changes in the patient’s health condition and interventions during transfer, ensuring this information is visible to the receiving facility.

Figures S1-S4 in Multimedia Appendix 1 include diagrams and illustrations detailing the workflows for viewing and updating patient data during transport requests and transfers. These figures demonstrate how critical patient information is initially shared with the receiving facility at the time of the transport request to ensure the appropriate ambulance and transport teams are dispatched to the patient. During transport, the EMS team uses a dedicated interface to document changes in the patient’s condition and any interventions performed. This information is visible in real time to the receiving hospital, enabling them to prepare effectively for the patient’s arrival (see Scenario and Use Case Diagrams in Multimedia Appendix 1).

#### Initialization: Input

A transport request is initiated by the sending hospital. A preconfigured job in the hospital’s database extracts the necessary patient data from its EHR system and securely stores them in a file on the hospital’s file system, which serves as an interoperability layer between the EHR and ITC-InfoChain.

#### Data Collection Isolation

Once the data are successfully collected, the file is disconnected from the EHR, ensuring separation of data collection from data extraction and recording on the distributed ledger.

#### Data Recording Process

The client program connects to the file and retrieves the encrypted patient data.

#### Blockchain Recording

Upon successful data extraction, the patient data are transferred to the HLF ledger. The data are encrypted and secured using public and private key cryptography.

#### Data Deletion

Once the data are successfully recorded on the blockchain, the original file containing the patient data is deleted to prevent any residual data from remaining on the file.

#### Output (Real-Time Access)

Enables staff in the critical care unit of the receiving hospital to view patient data in real-time, allowing them to determine the most suitable ambulance and transport team for optimal care, especially for critically ill patients. Paramedics can access these data before and during their journey to the sending facility, ensuring they are fully informed about the patient’s needs. During transport, the team can add a summary of any changes in the patient’s condition in real time, making this information immediately visible to the receiving facility. This capability allows the hospital to prepare the necessary equipment and clinical team in advance. If the patient’s condition requires specialized resources that the receiving facility cannot provide, the transport team can redirect the ambulance to an alternate facility equipped to handle the patient’s needs. Real-time data access during transport provides an essential layer of responsiveness, helping ensure that each patient receives timely and appropriate care.

#### Use Case Diagrams

For a more detailed explanation, refer to Multimedia Appendix 1, which contains use case diagrams illustrating key processes. Figures S5-S20 in Multimedia Appendix 1 depict these workflows, including retrieving and submitting transactions containing patient data to the network; querying patient data by the receiving facility’s critical care unit staff; adding new health condition information by the EMS transport team; and managing access control, identity verification, and certificate registration in the HLF network to ensure security, privacy, and confidentiality.

### Ethical Considerations

This research study did not involve any actual patient data. Only simulated data were used, which contained no identifying information linked to real individuals. Consequently, ethical guidelines related to participant privacy and data protection are not applicable to this study.

## Results

Refer to Multimedia Appendix 3 for viewing tables referenced in this section.

The ITC-InfoChain platform was evaluated in a simulated environment on AWS cloud infrastructure, where it demonstrated strong scalability and rapid processing times across different transaction volumes. Data were processed with varying batch sizes and transaction settings, allowing assessment of the platform’s real-time performance capabilities for interfacility transport scenarios.

Initial tests with a batch size of 50 transactions, a maximum number of transactions per batch set to 20, and a batch timeout of 1 second (the duration the system waits to fill a batch before processing it, even if the target size is not met) showed stable performance for lower transaction volumes, achieving an average processing time of approximately 3.1 seconds for 1 to 20 transactions and 6.4 seconds for 100 transactions (Table S1 in Multimedia Appendix 3). When the maximum number of transactions per batch was increased to 50 under similar conditions, transaction processing time remained stable, achieving approximately 3.1 seconds for lower transaction volumes and around 6.4 seconds for 100 transactions (Table S2 in Multimedia Appendix 3).

To enhance scalability, the system was configured with a larger batch size of 400 transactions and a higher transaction-per-batch limit of 100. This setup significantly reduced processing time for high transaction loads, achieving under 2 seconds for 400 transactions, demonstrating the system’s ability to handle extensive data input with minimal latency (Table S3 in Multimedia Appendix 3). Further testing with a transaction-per-batch limit of 200 confirmed sub-2-second processing times, highlighting ITC-InfoChain’s suitability for high-volume, real-time applications (Table S4 in Multimedia Appendix 3).

A comparative analysis of configurations is provided, demonstrating how increased batch sizes and message counts consistently reduced latency, achieving optimal performance for high transaction volumes, even in demanding scenarios (Table S5 in Multimedia Appendix 3). These results reinforce the importance of tuning parameters such as batch size, transaction per limits, and timeouts to achieve high throughput in HLF. Across configurations, results indicated that larger batch sizes and increased transaction-per-batch limits consistently yielded improved processing times, emphasizing the importance of tuning these parameters in HLF for high-throughput applications. The flexibility in system configuration allowed ITC-InfoChain to scale effectively, making it suitable for high-demand interfacility transport scenarios, where timely data access is critical for patient care.

Overall, ITC-InfoChain met expected performance targets, which is necessary for ITC at the time of transport request and during patient relocation.

## Discussion

### Summary of Main Findings

The study achieved its primary objective of developing and implementing the ITC-InfoChain platform, a blockchain-based solution designed to enable informed decision-making by accurately matching the appropriate ambulance to patients awaiting transport, all while ensuring EHR security and patient data privacy through an effective interoperability solution. This was accomplished by importing critical patient data at the time of transport request between the sending and receiving hospital. The platform’s architecture facilitated isolated DX from EHR systems, protecting patient privacy and EHR security while enabling real-time data accessibility for receiving hospital and transport teams, both during patient relocation.

The study also met its specific objectives, demonstrating the platform’s technical feasibility, performance, and reliability in securely isolating, recording, and importing patient data to the transport requests and keeping them accessible during the patient relocation. Key performance indicators confirmed that ITC-InfoChain achieved strong data transaction speeds, maintained reliability across various network configurations, and showed scalability under high transaction loads. These findings affirm the platform’s potential to enhance interfacility transport coordination, supporting timely, informed decision-making in patient care.

### Detailed Discussion of Findings and Comparison with Existing Literature

As a prototype for the interfacility transport scenario, ITC-InfoChain demonstrates a unique approach to managing privacy and security in high-stakes emergency data sharing. Unlike public blockchain models such as MedRec, which operates on Ethereum with broader access, ITC-InfoChain’s permissioned structure restricts data visibility strictly to authorized participants, a necessity in real-life interfacility transport where only select teams should access patient data. By isolating EHR data before integrating it into the blockchain, ITC-InfoChain prevents any direct access to these records during the transport request process, meeting data privacy and mitigating residual risks commonly associated with direct EHR interactions.

Real-time data access is essential in interfacility patient transport, where rapid decision-making can significantly prevent adversity during patient relocation. ITC-InfoChain addresses this need through the PatienTrack web application, enabling EMS teams and receiving hospitals to access patient data seamlessly during transport. Unlike previous solutions like MedRec and FHIRChain, which emphasize long-term data storage without prioritizing immediate access, ITC-InfoChain allows EMS and hospital staff to simulate quick, informed responses as they would in a real interfacility transport setting. Although HealthBlock includes some real-time features, its reliance on off-chain IPFS storage introduces latency, making it less ideal for time-sensitive patient transport care. ITC-InfoChain’s AWS-based infrastructure, on the other hand, ensures low-latency, high-throughput data access, giving paramedics and hospital staff the information they need for timely decision-making in critical transport scenarios.

Furthermore, ITC-InfoChain’s scalability demonstrates its capacity to handle high-demand situations typical in interfacility transport networks. Testing shows that the platform maintains quick transaction processing even at higher loads, like 400 transactions, confirming its suitability for complex transport networks requiring fast, reliable data access. Compared with other permissioned blockchain systems, such as FHIRChain and HealthBlock, which use multilayered architectures or off-chain storage that limits their real-time applicability, ITC-InfoChain leverages HLF to reduce transaction costs and avoids the costly consensus mechanisms found in Ethereum’s Proof of Work. This efficient, high-volume capacity suggests ITC-InfoChain’s strong potential for supporting scalable, sustainable applications in interfacility patient transport simulations and beyond.

### Limitations

This study has several limitations: First, communication and computational costs could not be accurately measured, as a long-term, fully operational version of the solution is required to assess these factors over an extended period. Second, this prototype was tested in a simulated environment, limiting the ability to observe real-world system responses, including data latency under varying network conditions across multiple health care systems. In addition, while the next phase will involve a pilot project using pseudonymized patient data from a regional hospital, the current study lacks data on how real-time operational demands may affect ITC providers’ decision-making. Another limitation is that, based on the complexity of each patient’s health conditions, the data extracted from the sending hospital’s EHR may vary, necessitating adherence to ITC protocols.

### Conclusions and Broader Implications

As a preliminary version, ITC-InfoChain successfully demonstrated its functionality, scalability, and feasibility in supporting secure, real-time data sharing for interfacility transport by addressing key concerns around data privacy, EHR security, and interoperability challenges. Initial feedback from the head of a critical care unit in a regional receiving hospital was positive, highlighting the platform’s potential to enhance patient transfer services. However, resistance from some stakeholders, due to unfamiliarity with blockchain technology and persistent data privacy concerns, underscores the need for further engagement. A pilot program is sought to evaluate ITC-InfoChain’s real-world impact, address stakeholder concerns, and provide targeted education to facilitate broader acceptance.

## Appendix

Multimedia Appendix 1 Supplementary content and figures.

Multimedia Appendix 2 Checklist for the Reporting on Digital Health Implementations.

Multimedia Appendix 3 Supplementary tables.

## Abbreviations

API: application programming interface
AWS: Amazon Web Services
DX: data exchange
EHR: electronic health record
EMS: emergency medical services
HIO: health information exchange organization
HIPAA: Health Insurance Portability and Accountability Act
HL7 FHIR: Health Level Seven Fast Healthcare Interoperability Resources
HLF: Hyperledger Fabric
IPFS: interplanetary file system
ITC: interfacility transport care
SDK: software development kit
SNOMED CT: Systematized Nomenclature of Medicine–Clinical Terms

## Data Availability

This study used simulated data, and no actual patient data were used. The simulated datasets generated and/or analyzed during this study are available from the corresponding author upon request.
