# Supplementary material for: Appropriately Matching Transport Care Units to Patients in Interhospital Transport Care: Implementation Study
Source: JMIR Form Res. 2024 Dec 13;8:e65626. doi: 10.2196/65626 (PMC11681279; doi:10.2196/65626)
Supplement: Multimedia Appendix 1 [file formative_v8i1e65626_app1.docx]

The following subsections extend their main sections, providing detailed explanations and supporting images.

Extension of Method System components

In the architecture of ITC-InfoChain, a critical component that integrates patient data into the blockchain network is the Client Program. This program plays a key role in initiating the transaction process by reading patient data from a file in the sending facility’s system and interacting with the blockchain. Here is how the Client Program is embedded into the overall workflow, connecting various components of the ITC-InfoChain system:

# Client Program

The Client Program is responsible for reading patient data from the shared file stored in the sending facility's file system or on the cloud (AWS) based private infrastructure. This file contains patient information collected from the EMR system (e.g., Cerner) at the sending hospital and disconnect from it. The Client Program acts as the gateway between the sending hospital’s internal system and the blockchain network. It performs the following key functions:

**Reads patient data**: The program extracts patient information from the file, which temporarily holds the patient’s critical medical data before it is transmitted to the blockchain.

**Connects to a Peer Node**: Once the data is retrieved, the Client Program connects to a peer node on the network, representing the sending hospital, and prepares the data for blockchain submission.

# Peer Node

In the ITC-InfoChain system, each hospital is represented by its own peer node, which can be deployed on a local server or in a private cloud. A peer node in a Hyperledger Fabric (HLF) blockchain network, such as ITC-InfoChain, represents an essential technical component in the system. When we say a peer node "represents a hospital," we are referring to a software package residing on a virtual machine (VM), that interacts within the blockchain network on behalf of that hospital. This way, the peer node represents a hospital as a member of the network. This node handles requests for data, processes transactions, and ensures the integrity of the ledger for that hospital

**Transaction Proposal Creation**: The Client Program formats the patient data as a transaction proposal (TX) and submits it to the peer node.

# Chaincode Execution:

The peer node then communicates with the chaincode (smart contract), which contains the business logic for handling patient data. The chaincode processes the transaction by writing the patient's data to the ledger, which consists of both the blockchain (for historical record) and the state database (for the current patient data). The chaincode updates the blockchain ledger by recording the transaction in a new block, and the state database stores the most up-to-date patient information.

# Ledger: Blockchain and State Database

The ledger in the peer node comprises two parts:

**Blockchain**: This stores the entire history of transactions in an immutable format. Each transaction is written as a block, and the blocks are cryptographically linked to form a chain, ensuring the integrity and security of patient data. The new transaction (TX) created by the Client Program is appended to the blockchain after being processed by the chaincode.

**State Database** (e.g., CouchDB): While the blockchain stores historical data, the state database keeps track of the most recent state of the patient's data. For example, if the patient's condition or treatment plan is updated during the transfer, this information will be reflected in the state database for quick access and real-time querying by the receiving hospital.

# Orderer Nodes: Block Creation and Distribution

After the peer node processes the transaction, the endorsed transaction is sent to the orderer node. The orderer node plays a pivotal role by:

**Creating New Blocks**: The orderer node orders the transactions chronologically into blocks.

**Distributing Blocks**: It then disseminates the newly created blocks to all relevant peer nodes across the network, ensuring that each peer node’s blockchain ledger is updated consistently.

# PatienTrack Web App and Receiving Facility

The PatienTrack web app is the interface that allows healthcare providers to interact with the blockchain system. At the receiving hospital, the PatienTrack app queries the peer node for patient data:

# Accessing Patient Data:

As the transaction has already been written to the blockchain and the state database by the sending hospital’s peer node, the receiving hospital can access up-to-date patient

information. The PatienTrack app enables medical staff at the receiving facility’s critical care unit to retrieve the latest patient data, including any updates made during the transfer. The web app also allows the relocation team to add a care summary during the transfer, updating the receiving facility between the acceptance and completion of the transfer, which aligns with the third pillar of best practices for interfacility transfer [1]. We build the system on a cloud-based HLF peer-to-peer network of all hospitals and emergency services involving Interfacility Transport Care (ITC) for scalability, performance, and cost- effectiveness

# Data Writing Process

Procedure: Upon receiving a TX from the client program, the peer node sends it out to the chaincode. The chaincode interacts with the state database (DB), reads the patient's existing data and creates a read set. It performs a process on the read set with the data received from the TX, creating a write set that is supposed to help update the State DB and verify the TX recording in the block after the consensus mechanism is successfully completed. It means all designated peers need to follow the same process and report their read & write sets to the client program. If the client program finds out that all sets are identical, it signs the original TX and sends it out to the ordering node to apply the consensus protocal before disseminating the TXs/blocks to the peers that update the StateDB and commit the blocks. For example, if existing data on the State DB shows 20 ml of the medicationA and the TX request is 30 ml, then the read set shows (medicationA: 20) and the write set would be (medicationA: 30). While the State DB will save only the latest data (medicationA:30), the blockchain will save both data as part of the TXs in the block, hence the TX history.

# Endorsement policy enforcement:

Objective: Ensure transaction validity and consistency across the network.

Procedure: The endorsement policy is part of the writing process explained earlier. The client program SDK implements the endorsement policy by matching all read and write sets reporting by the designated endorsing peers and ensuring that they are identical.

# Transaction ordering:

Objective: Ordering and validating the transactions.

Procedure: The client SDK forwards endorsed transactions to the leader orderer node. Using the Raft consensus algorithm, this node sequences the transactions, constructs blocks, and sends these to follower orderer nodes for validation and acknowledgment. Once validated, the blocks are disseminated to the peer nodes.

# Consensus mechanism Security:

Objective: Secure and stabilize the consensus mechanism.

Procedure: HLF employs protocols like the peer-to-peer gossip protocol to ensure synchronization and consensus among nodes, even if some nodes do not receive the blocks initially.

# Transaction validation and commitment:

Objective: Confirm transaction validity and update the database.

Procedure: The peer nodes validate transactions, appending them to their respective blocks and updating their State DBs. This process ensures that transactions are legitimate and securely recorded on the blockchain.

# Crash tolerance:

Objective: Maintain network functionality during ordering node failures.

Procedure: The Raft consensus protocol, characterized by its Crash Fault Tolerance (CFT), provides resilience to the network. It allows the network to function even if some nodes fail. The number of crashes a Raft cluster can endure depends on its configuration. Various features in the Raft protocol, like leader election timeouts and heartbeat mechanisms, allow it to adapt to different network conditions and minimize the impact of node failures [2].

# The extension of Interoperability and Data access in the manuscript

**Data access at the time of Transport Request Service**

Figure S1 shows the patient's information to the receiving facility at the time of the Transport request. The information is crucial for the receiving hospital to prepare adequately for the incoming patient and provide continuous care.


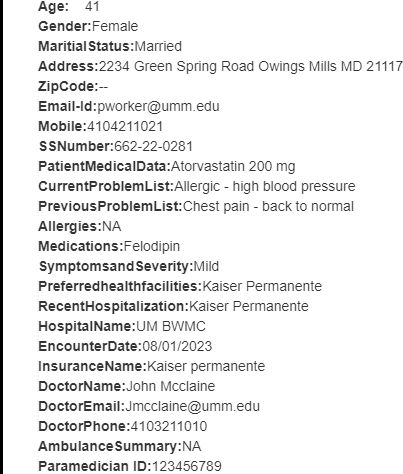


Figure S1: Patient information provided to the receiving hospital at the time of the transport request

After reviewing the patient's data, the hospital will decide if its transport unit has the necessary infrastructure and expertise for the transfer. This is vital for ensuring the patient's safety and

proper care during transport. If the receiving facility accepts the patient, their in-house transport team is dispatched to the sending facility, fully prepared based on the patient's data. In cases

where the in-house team is unavailable or lacks the necessary resources, an external EMS may be called in.

- **Non-integrated EMS**: Receives only essential patient data.
- **HLF-integrated EMS**: Can access detailed patient data using their license number, patient name, and phone number.

Both the in-house team and external EMS can access and update patient data in real time via the

**PatienTrack mobile app**. They can also add a care summary during transport, which is immediately visible to the receiving facility's critical care unit through the patient portal.

Figure S2 shows the interface where the relocation crew can add a care summary. This process improves communication, preparedness, decision-making, and compliance with medical

protocols, while enhancing patient care and efficiency.


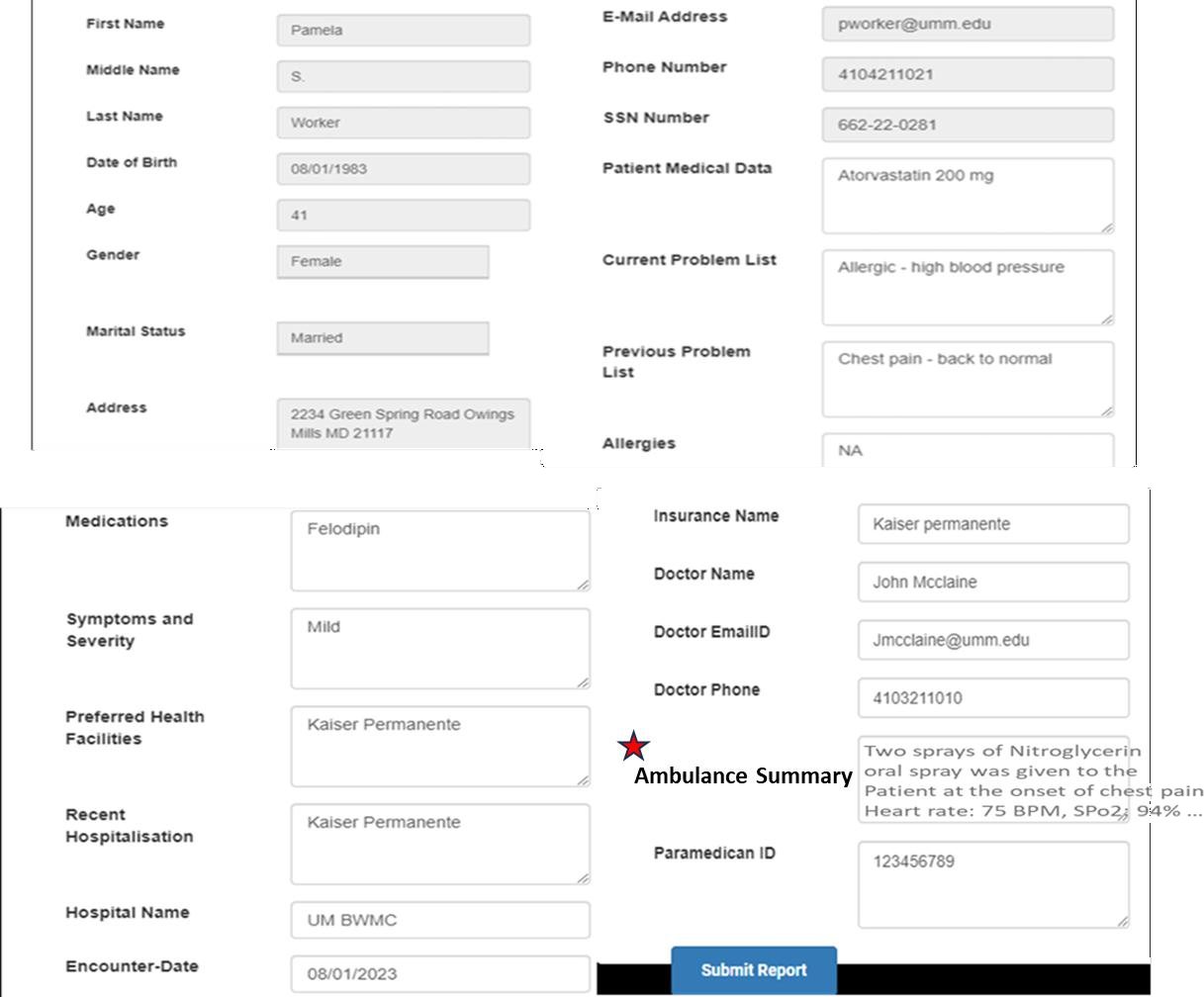


Figure S2: Interface used by the relocation team to input new data on patient condition changes and interventions during transport

The transactions (TXs) and patient data become visible in the receiving hospital after the EMS submits them. By clicking 'view,' as shown in Figures S3 and S4, the new patient information and intervention summary can be accessed.


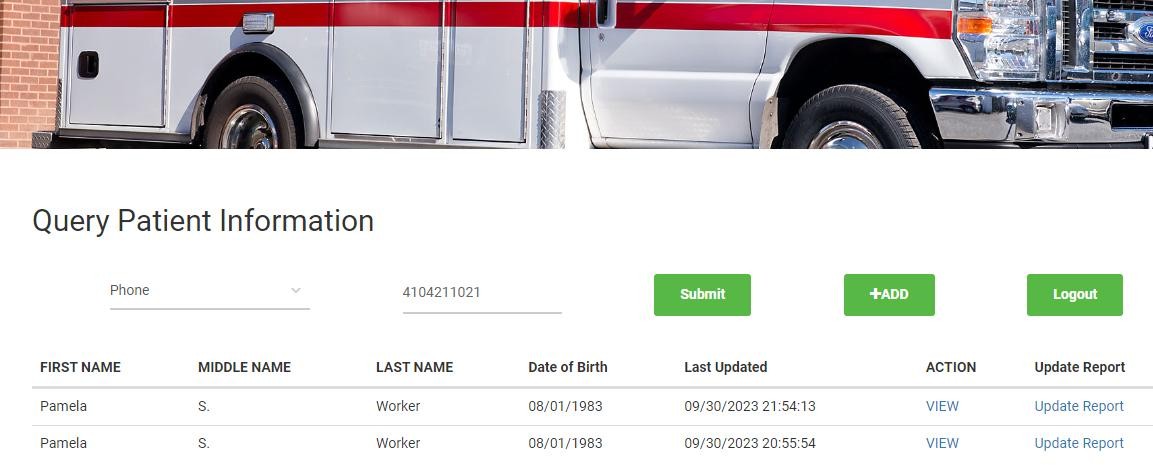


Figure S3: Display of patient data and intervention summary submitted by the EMS team, accessible to the receiving hospital during transport


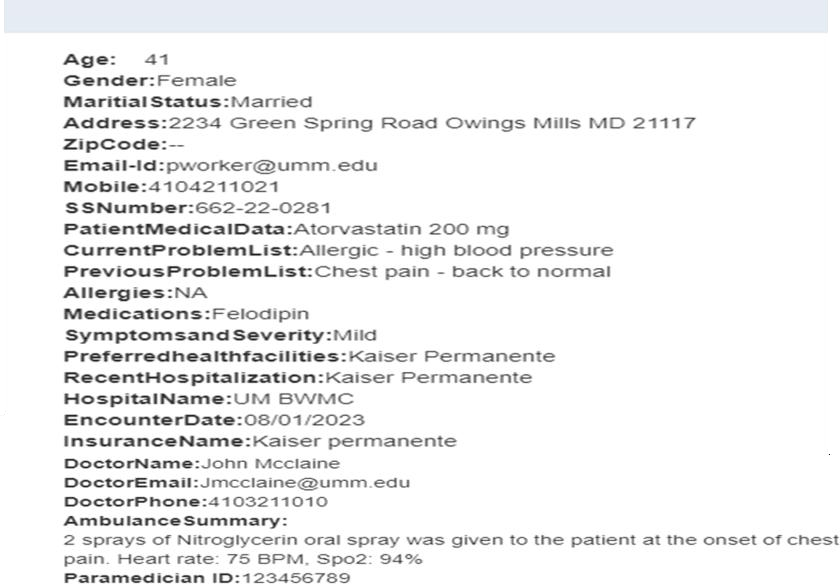


Figure S4: Detailed view of patient data and intervention summary submitted by EMS, accessible to the receiving hospital during transport

While Figure S1 shows the initial patient information provided at the time of the transport request, Figure S4 includes additional ambulance intervention details, such as administered treatments, that are absent in Figure S1.

# Extension of Scenario and Use case diagrams

Figure S5 illustrates the interactions between the client program and HLF network components for recording patient's data on the ledger


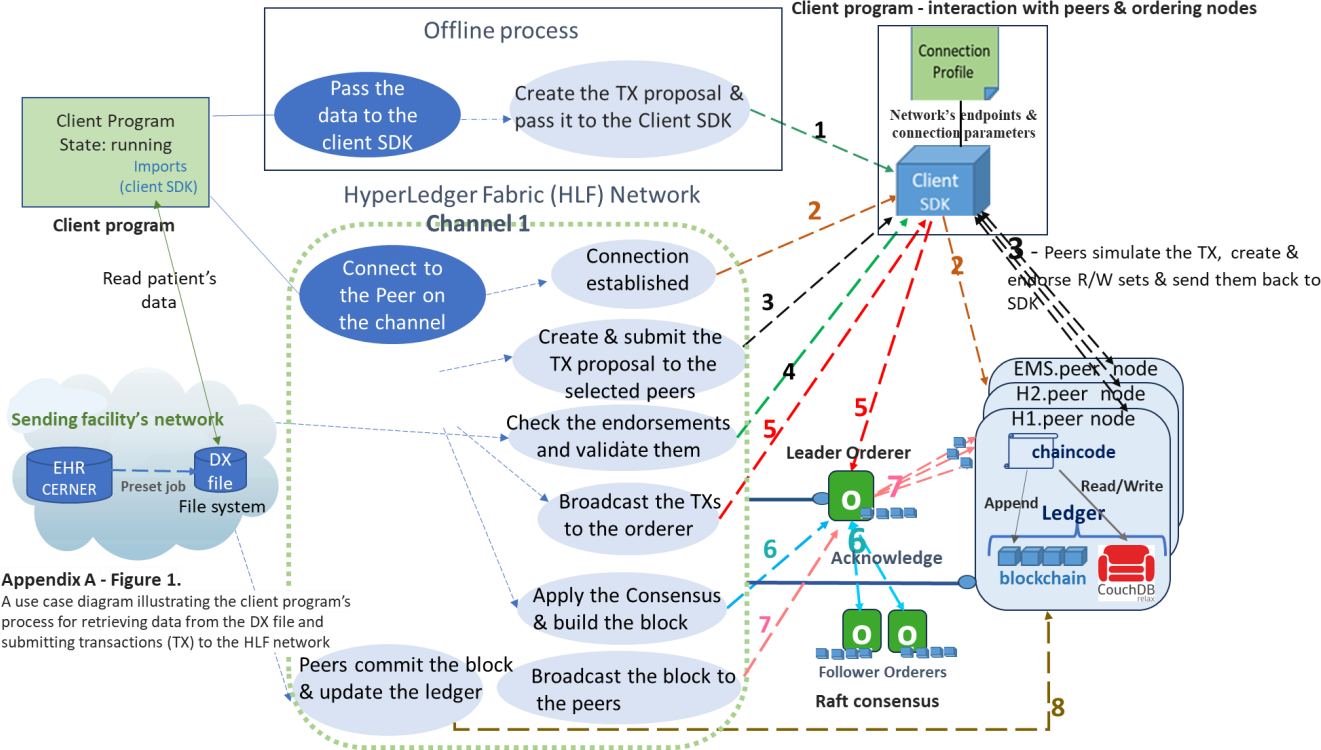


Figure S5: A use case diagram illustrating the client program’s process for retrieving data from the sending hospital file system and submitting TXs to the HLF network

Figure S6 depicts the interactions between the client program and HLF network channel components for querying patient's data from the ledger.


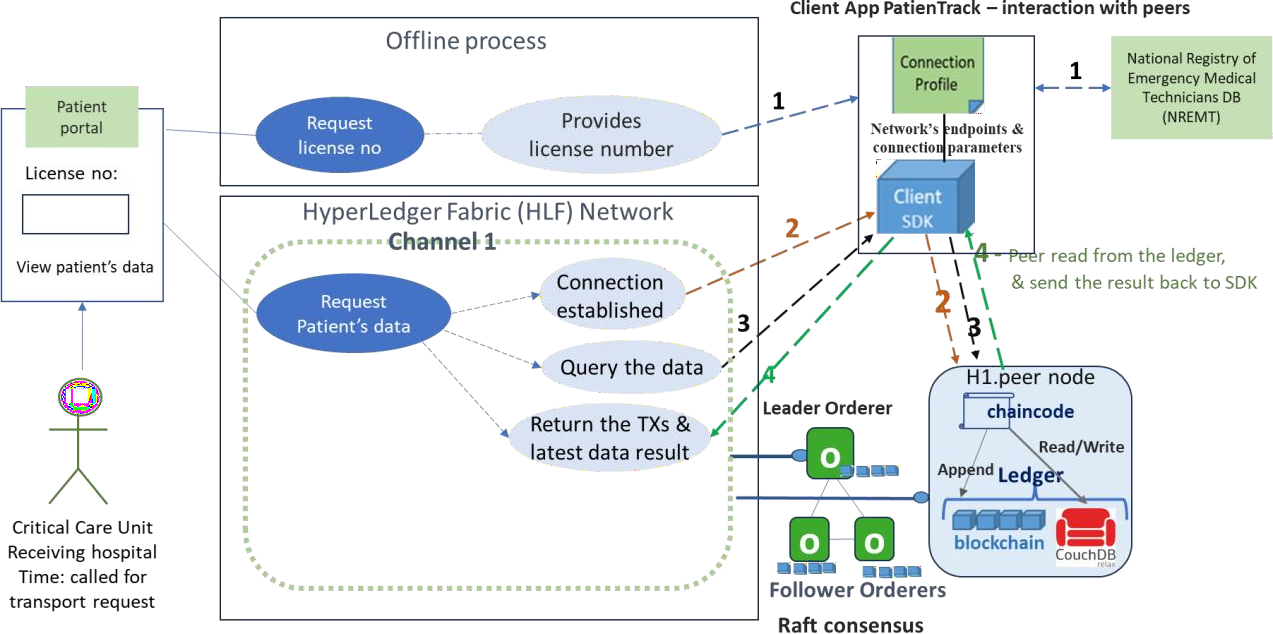


Figure S6: Illustration of the process by which critical care staff at the receiving facility query patient data from the ledger


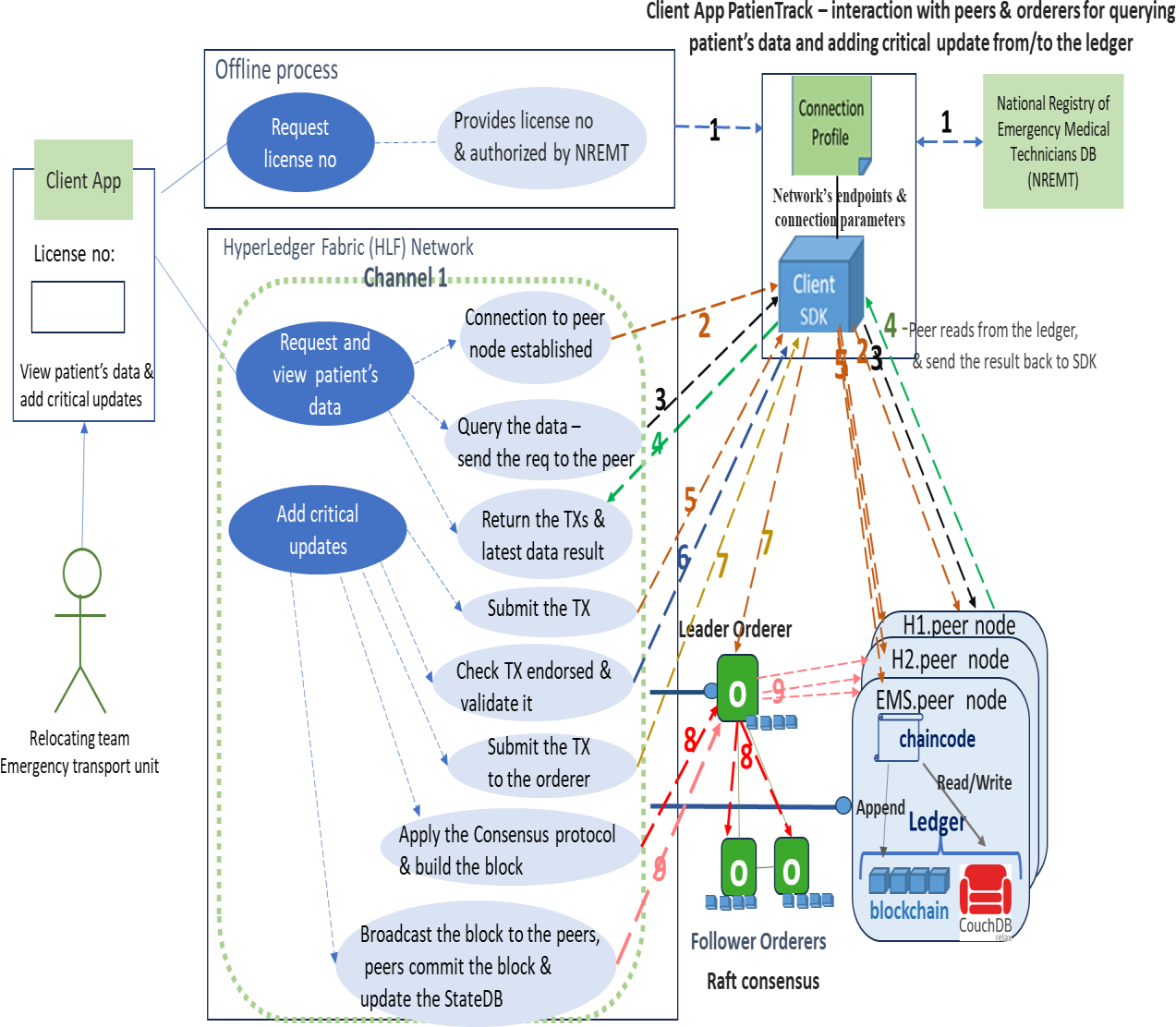


Figure S7: Diagram showing the EMS transport team’s interaction with the ledger for querying and adding patient care summaries

# Security, privacy, and confidentiality of the permissioned blockchain HLF

The consensus mechanism is crucial for ensuring the integrity, security, and reliability of the blockchain network. It plays a pivotal role in achieving agreement among network participants, validating TXs, and maintaining the ledger's immutability in a permissioned and efficient manner. However, the network's resilience against cyber threats is not solely dependent on the consensus mechanism. Additional security features and customized configurations also contribute significantly to safeguarding the system.

Marc Andreessen, a prominent figure in Silicon Valley, has described the blockchain's distributed consensus model as the most important invention since the advent of the Internet. HLF demands stringent measures such as identity management, accountability, access control, and authorization to establish a reliable platform. Moreover, it mandates data encryption during all communications, employing the Transaction Layer Security (TLS) protocol to provide an additional layer of security.

# Members (Hospitals) Identity management

In use case diagrams 3, 4, and 5, various actors—including peers, ordering nodes, and client program (admins and users)—participate in the TX lifecycle. Each actor requires two certificates and a private key to engage in transactions.

During registration, admins issue a private key to the organization's users. Admins can enroll users, or users may enroll themselves to obtain an Ecert (Enrollment Certificate) and a TLS (Transport Layer Security) certificate. The Ecert serves as an identifier during transactions, containing crucial information like a public key, issuer's name, the issuer's Ecert, etc. The TLS certificate, comprising a pair of public and private keys, facilitates secure communication and message encryption between actors.

A sender actor uses the private key to sign a transaction, authenticating themselves as a trusted source to the recipient. The recipient then derives a public key from the signature, comparing it with the sender's public key to verify the transaction's origin. Since the HLF channel's membership service provider (MSP) initially contains the public keys associated with all actors (created using actors' Ecert and TLS certificates before the channel's creation), any actor can access these keys. When new peers or users join the network, the channel MSP is updated to include them. Figure S8 illustrates actors exchanging certificates during transactions.


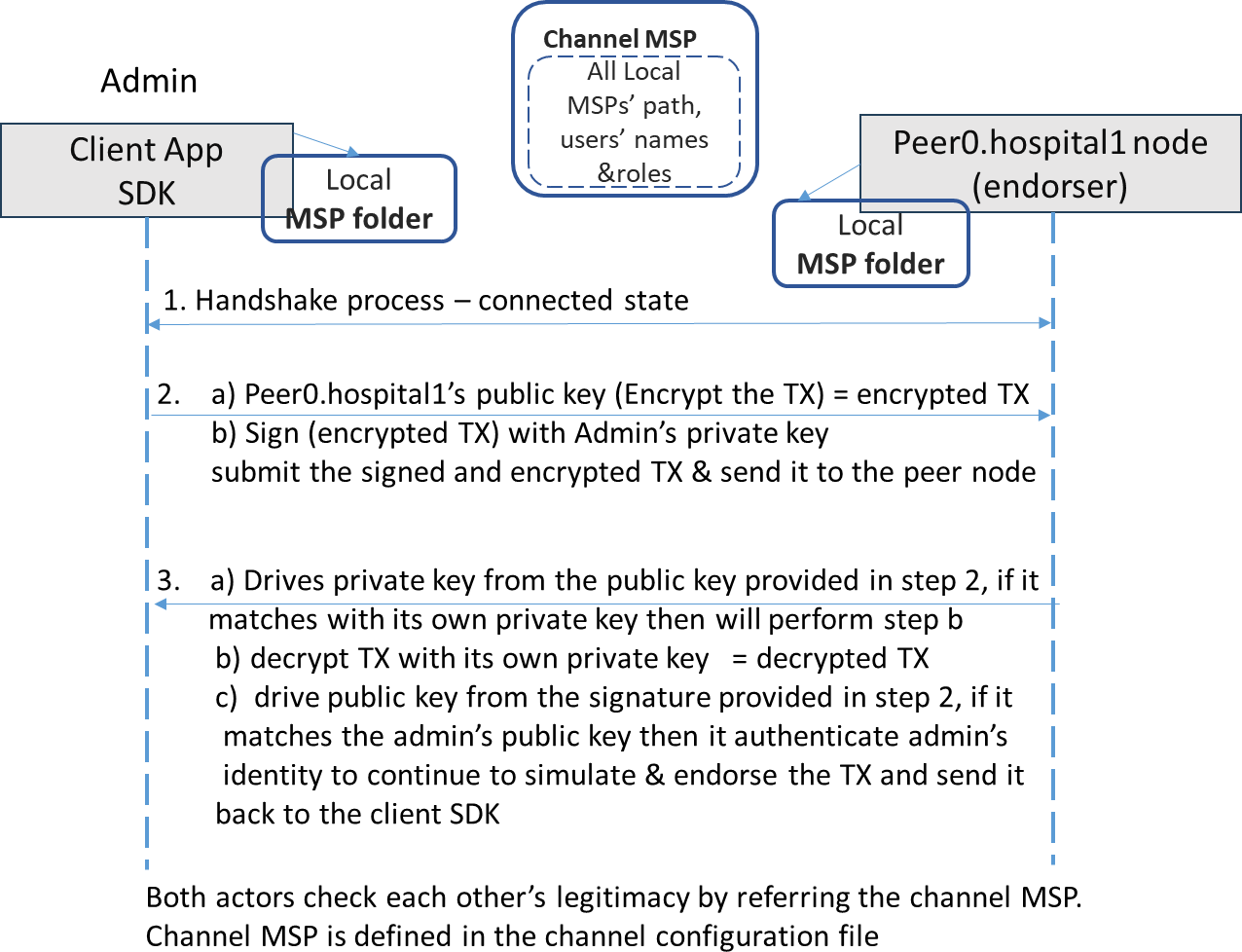


Figure S8: Interaction between the Client program’s SDK API and the peer node while exchanging certificates

The Client program’s SDK component through its in-built APIs, initiates a handshake to connect with the peer node. To achieve this connection, the program must incorporate the HLF Client SDK into its code. Various SDKs are available for different programming languages, each providing APIs for certificate submission and transaction (TX) initiation. These SDKs utilize a connection profile, essentially a configuration file that introduces the HLF network to the Fabric Client SDK [3-4].

During a transaction, the client program encrypts the TX proposal using the peer node's public key, signs the encrypted proposal with its private key, and then sends it to the peer node. Upon receiving the proposal, the peer node uses its private key to verify and decrypt the TX proposal. It then extracts a public key from the received signature for client program authentication, ensuring the signature originates from a trusted source. This process uses the admin user certificates for legitimacy [5].

A channel MSP instance is accessible to all network components, allowing them to use or verify each other's public keys throughout a TX lifecycle. For instance, a peer node can extract a public key from a signature sent by the client SDK and match it with the available public key from the channel MSP. This system also supports encryption using each other's public keys available through the channel MSP, with roles, rules, and channel policies being verified during the TX process.

Security is paramount, and the private key is critical for signing transactions. If it is compromised, the network's integrity is at risk [3]. Therefore, it is advisable to store the private key securely, away from Local MSPs, preferably in a hardware module on a machine [5]. This practice enhances

network security by reducing the risk of private key exposure. It is worth noting that HLF V2.5, an upgrade from the HLF V2.2 used in this paper, offers additional features that enhance privacy, performance, and channel maintenance [6].

# Certificate registration and enrollment process

The Fabric Certification Authority (CA), a Hyperledger Fabric (HLF) component, is a server software package that is containerized for ease of deployment and scaling. Its primary role is to issue digital identities to users within member organizations as well as to various fabric components like peer nodes, ordering nodes, and others. Administrators interact with Fabric CA using a tool called 'fabric-ca-client', a binary utility designed to facilitate user registration. This process connects to a type of CA known as a Root CA (RCA) [7]. It is crucial to note that HLF provides flexibility regarding the use of Certification Authorities. Depending on the network's design and security needs, these authorities can be either internal entities within the organization or external, third-party organizations [8].

Figure S8 visually represents the process through which RCAs and Intermediate CAs (ICAs) issue certificates to organization users, and a similar process is also applicable for peer and ordering nodes.


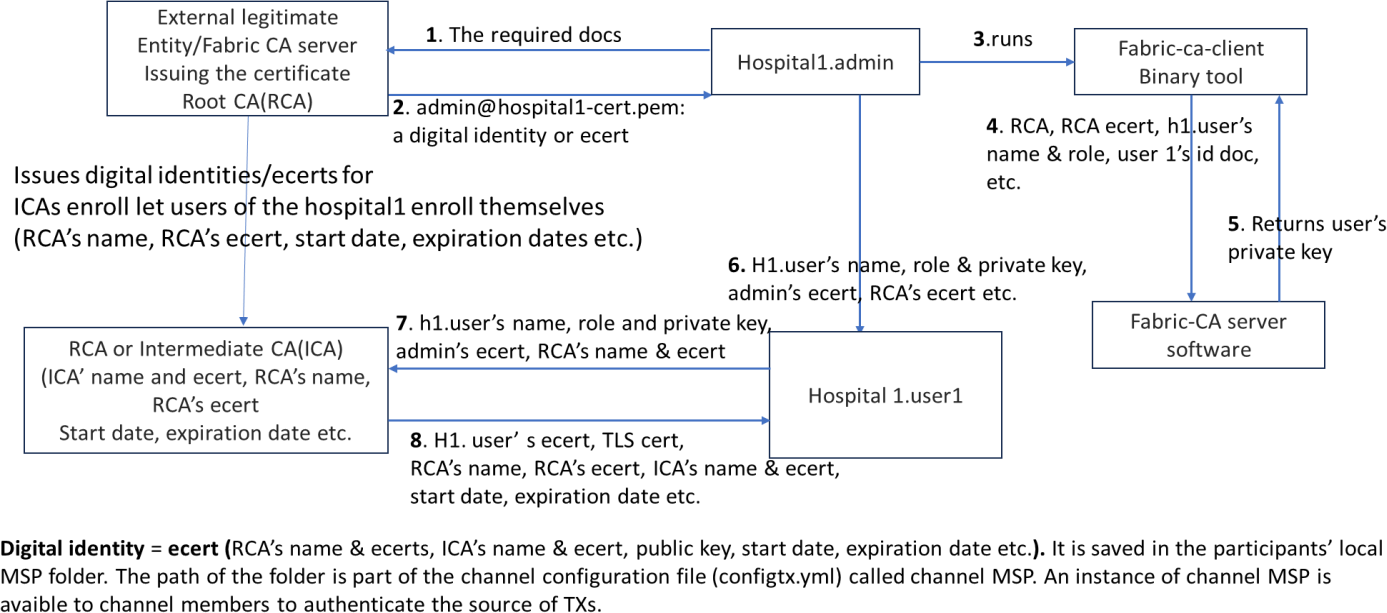


Figure S9: Roles of RCAs and ICAs in the registration and enrollment process for all participants, including organizational users, peers, and orderers

Joining a channel within the HLF network requires organizations and their users to possess

digital identities or certificates issued by a trusted entity. Without these, an organization cannot participate in the channel. The channel's MSP must incorporate the relevant cryptographic information into the channel configuration file (named 'configtx.yaml') to facilitate this. Without this step, transactions initiated by the organization's identities will be rejected, as the Channel MSP, accessible to all channel members, will not recognize them [6]. The Local MSPs, created

during enrollment, are hierarchical structures holding certificates (Ecerts and TLS certs) for users

and HLF components. These are established before creating or updating a Channel MSP. In this paper, the terms 'actors', 'participants', and 'HLF components' are used interchangeably.

The CA plays a crucial role in managing certificates for network participants, and overseeing their issuance, renewal, and revocation [9]. Typically operated by the administrator of a trusted network member, the CA ensures secure and authenticated participation. A reliable external CA should validate the certificates for admins. In some setups, an admin registers organization users who can then enroll themselves to generate their certificates. This process maintains user privacy, preventing admins from accessing users' identities [10].

Figure S9 illustrates the registration and enrollment process through a sequence diagram detailing the interaction between the 'Fabric-ca-client' and 'Fabric-CA server' components to obtain certificates.


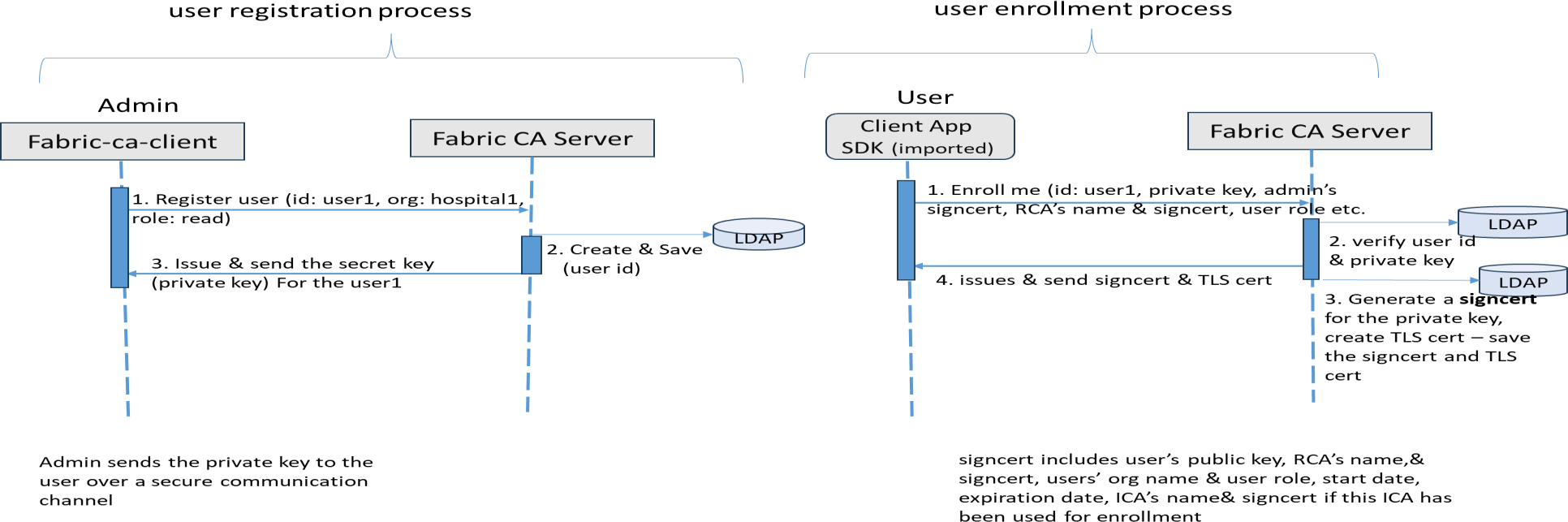


Figure S10: Sequence diagram illustrating the registration and enrollment process for network members (hospitals) to obtain certificates

During enrollment, the CA server software establishes a hierarchical folder system for each actor in HLF. This system includes subfolders such as 'msp' and 'tls', where the private key and

Enrollment Certificate (Ecert) are placed within the 'msp' folder. In a production environment, the private key would be stored in a different, secure location, not within the 'msp' folder.

Figure S10 shows how a client SDK uses administrator certificates to communicate with peer

nodes. The paths to the 'msp' and 'tls' folders must be specified in the client program’s SDK code to enable certificate-based connections and communication with peer and ordering nodes. In the test environment described, the private key is stored within the 'msp' folder, but in a production

setting, it would be moved to a more secure location. Figure S11 displays the subfolders contained within the 'msp' folder.


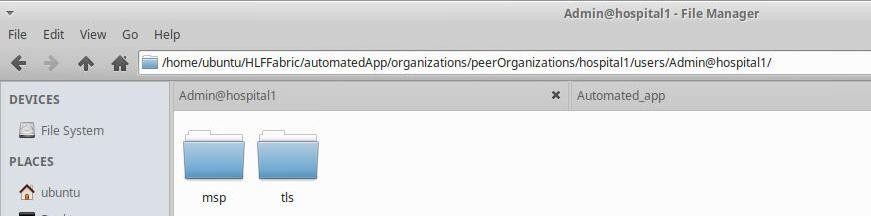


Figure S11: Structure of the 'msp' and 'tls' folders created during the enrollment process, containing

essential cryptographic materials used to sign the TXs submitted by any entity inside and outside of the network


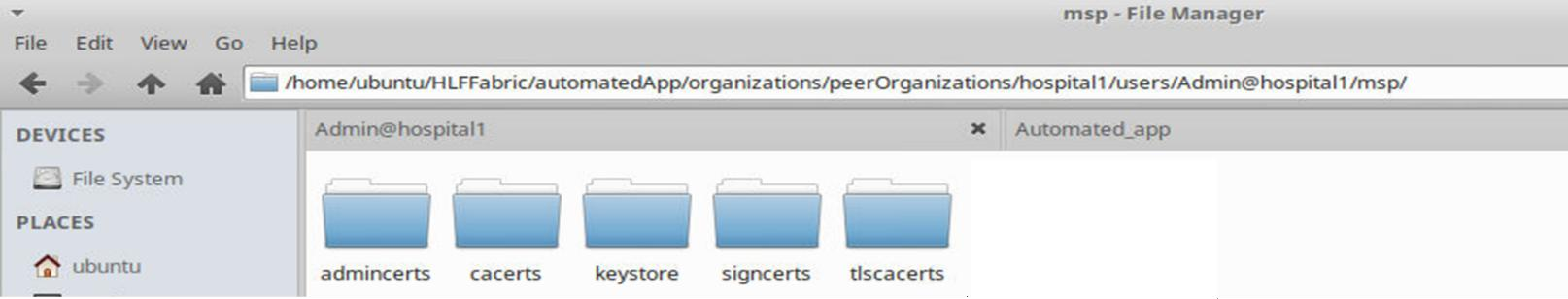


Figure S12: Contents of the keystore folder, including the admin’s private key and other necessary certifications

Figure S12 shows the keystore containing the admin's private key for signing transactions via the client SDK. Peer nodes use this signature to extract and compare the admin's public key with the one in the Enrollment Certificate (Ecert) on the channel MSP to validate authenticity. Figure S13 displays the Ecerts folder holding the admin's digital identity.


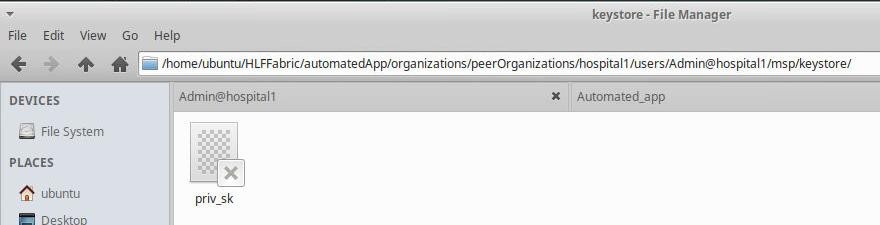


Figure S13: The private key of the hospital admin that is used to sign the TXs


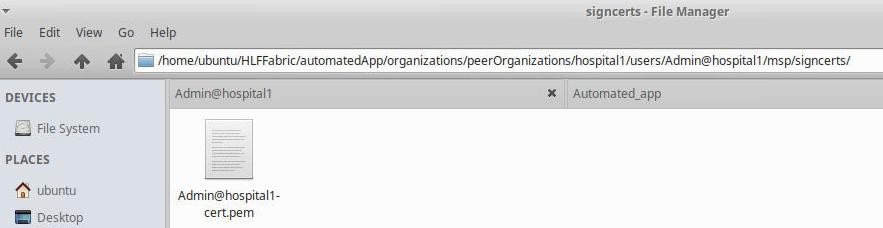


Figure S14: Admin's enrollment certificate (Ecert) used for matching with the sender's digital signature to verify authenticity

Note that the paths in the Channel MSPs, which hold all Ecerts for channel members, are accessible to all participants, as indicated in Figure S13. Members securely hold their private keys, each associated with a public key listed in the signing certificate. These private keys are crucial for signing transactions and performing other significant cryptographic operations.

When initiating a transaction, a member signs it using their private key. This signature, along with other essential information (such as the proposal response, endorsement policy, and transaction request), is incorporated into the transaction proposal, which is then sent to peer nodes for endorsement. Peer nodes use the public key in the Ecert to verify the signature's validity. If the signature is confirmed as valid, the peer nodes move forward to simulate and endorse the transaction.


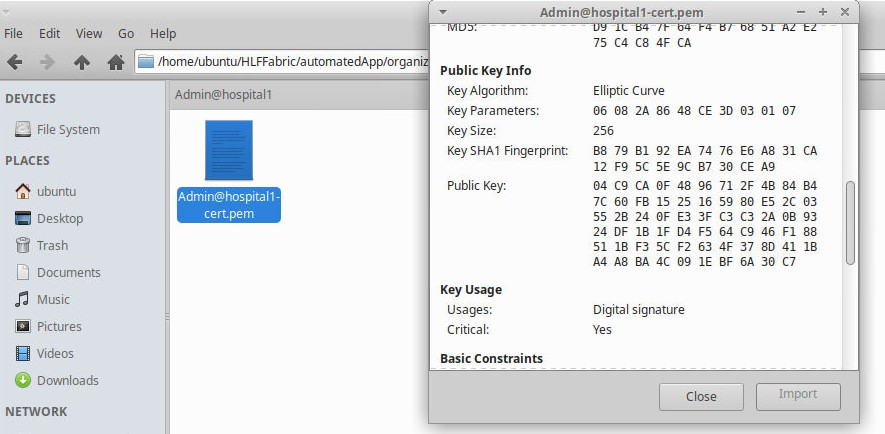


Figure S15: Details of the information embedded within an enrollment certificate (Ecert), including public keys and issuer data


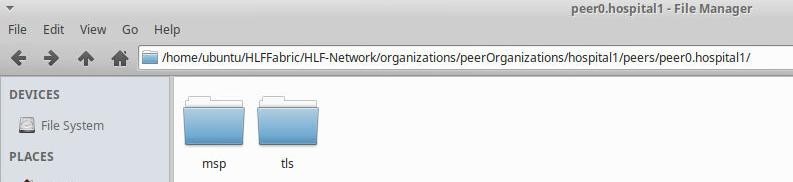


Figure S16: Folder structure created by the Fabric CA server software, showing the MSP and TLS directories for peer nodes

It shows the same crypto materials for the peer nodes that participate in TXs process.


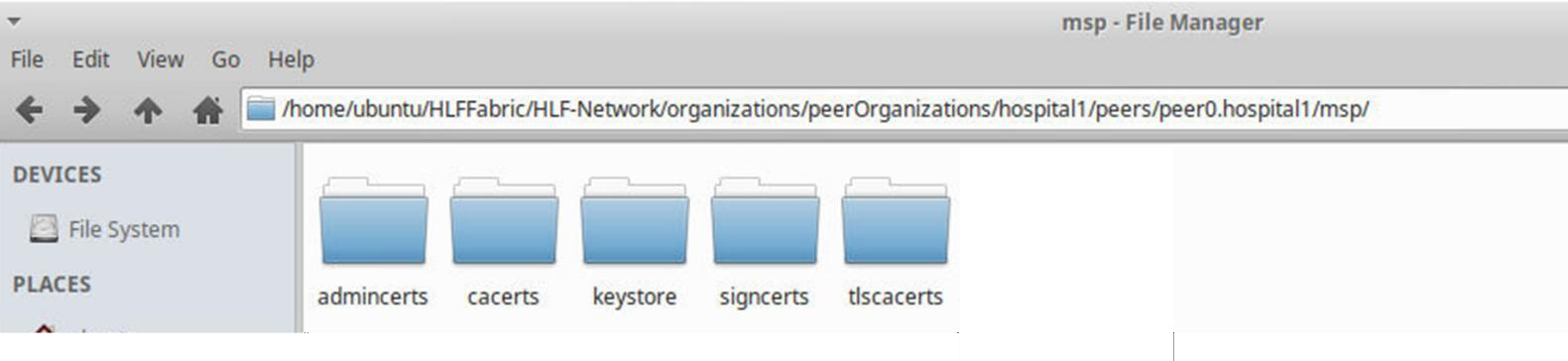


Figure S17: Folders created by the CA server for storing the private key and enrollment certificates (Ecerts)


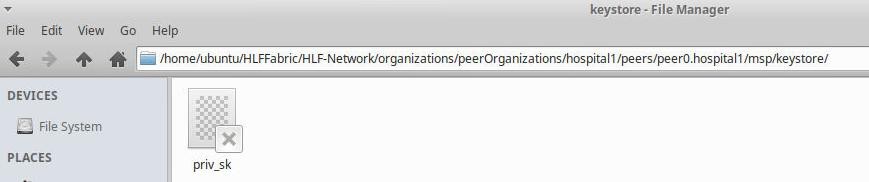


Figure S18: The private key that the peer node (representing hospital on the Network) uses to sign the TX.


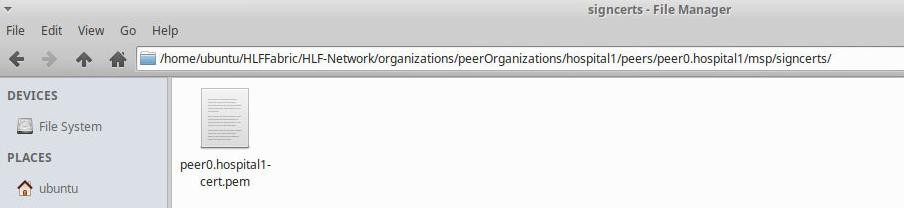


Figure S19: Enrollment certificate (Ecert) of the peer node named 'peer0-hospital1-cert.pem' for authentication in the HLF network

Based on Figure S18, the Ecert of the peer node includes a public key that is distributed within the channel, allowing other participants to authenticate the peer node using the signature provided by its private key. Figure S19 shows the contents of the TLS folder.

Impersonating a public key is almost impossible because if a hacker attempts to impersonate a sender and send a transaction (TX) using the receiver's public key, they must sign the TX with

their private key. The receiver verifies the sender’s identity by checking the signature against the public key stored in the channel MSP, which contains all identities, identity issuers, roles, and

public keys. These identities are part of the channel configuration file, which is configured only by the selected admins of the most trusted members. The identities stored in files can be accessed in the channel MSP via their paths.


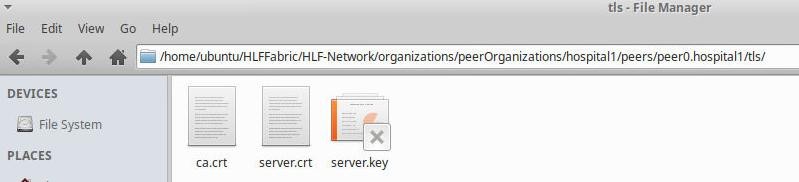


Figure S20: TLS cryptographic materials used for secure connection and message encryption in the network

As illustrated in the figures, a peer node intending to send an endorsed TX to the client program’s SDK must first locate its TLS certificate (server.crt) within its local MSP folder. This certificate is used to encrypt the TX for secure communication before it is sent to the client program’s SDK. The peer node then signs the encrypted TX with its private key (server.key) to authenticate itself. The client program’s SDK, which has access to the peer node's public key

(peer0-hospital1-cert.pem) through the channel MSP folder, can then verify the peer node's

identity. Initially, the client program decrypts the message using its TLS private key, and then it authenticates the peer node by retrieving the peer node's public key. The path to this public key in the channel MSP is provided within the code. After these verification steps, the peer node

proceeds to simulate the TX, taking into account various other pieces of information such as RCA names, Ecerts (digital identities), ICA names and Ecerts (if used during the enrollment process), start dates, expiration dates, etc.

Similarly, a client program’s SDK refers to its own MSP and TLS folders to find the necessary cryptographic materials. It uses the admin.hospital1-cert.pem to encrypt the TX and the priv_sk to sign it. The MSP in HLF is pivotal in identity management, authentication, and authorization within a permissioned blockchain network. It recognizes network participants and assigns the appropriate permissions for network interaction. The MSP translates identities into roles, defines organizational units (OUs), and establishes structures for channel usage, thereby playing a vital role in the network's security and functionality. The system employs public key infrastructure- based asymmetric encryption and digital signatures to safeguard shared patient data.
