# Supplementary material for: Appropriately Matching Transport Care Units to Patients in Interhospital Transport Care: Implementation Study
Source: JMIR Form Res. 2024 Dec 13;8:e65626. doi: 10.2196/65626 (PMC11681279; doi:10.2196/65626)
Supplement: Multimedia Appendix 2 [file formative_v8i1e65626_app2.pdf]

Study Design: Digital health implementation

Reporting guidelines on Digital Health Implementations

iCHECK-DH: Guidelines and Checklist for the Reporting on Digital Health Implementations

https://www.jmir.org/2023/1/e46694

Item 1: Title (M)

Identify as an implementation report and describe the implementation in the title, keywords, or both.

Examples

- "Chatbot-Based Assessment of Employees' Mental Health: Design Process and Pilot Implementation" [18]
- "Creation and Global Deployment of a Mobile, Application-Based Cognitive Simulator for Cardiac Surgical Procedures" [19]
- "The Journey to National Scale of Zanzibar's Digitally Enabled Community Health Program: An Implementation Report" [15]

Explanation

Authors should choose a title that is concise yet accurately describes the purpose of the document as an implementation report to ensure maximum visibility and easy access. Strategic use of keywords in the title can make this document searchable in various publication databases for optimal reach and machine processing, without the need to explicitly mention "implementation."

Answer:

Title: Appropriately matching transport care units to patients in an interhospital Transport Care: an implementation report

main objective: matching transport care units and transport team to patient needs
setting: interfacility transport care
purpose: importing patient data into transport requests

Item 2: Abstract (M)

Provide a summary of the key elements of the implementation report, including a description of the implementation strategy and the intervention, defining the key elements of the implementation and health outcomes, and specify the key performance indicators (KPIs)/outputs.

Example

- "Background: Patient empowerment can be associated with better health outcomes, especially for chronic diseases. Concerto is a mobile application designed to promote patient empowerment in an in-patient setting. Methods: The application was designed and prototyped during a hackathon. It uses data from the hospital information system to provide key functionalities: a care plan, access to targeted medical information, practical information, information about the nursing team on duty, and a medical round preparation module. Following a feasibility study, funding was obtained, and the application developed using an agile methodology and deployed in 4 pilot divisions, using institution-owned iPads. Results: The project lasted for two years with effective implementation in the 4 pilot divisions, within budget. The induced workload on caregivers was a key challenge that warranted a change in our implementation strategy. The presence of a killer-function would have facilitated the deployment. Furthermore, our experience is in line with the well-accepted need for both a high-quality user-training and a good selection and engagement of super-users. By presenting Health Information System data directly to the patient, Concerto has highlighted them to be not fit-for-purpose and has triggered data curation initiatives. Finally, connecting the application to the HIS has promoted the usage of standards, both on the HIS and mobile applications side, that should facilitate future initiatives. Discussion: This implementation report presents a real-world example of implementing a patient-empowering mobile application in an in-patient setting of a University Hospital. One limitation of the study is the lack of definition of a Key Success Indicator" [16].

Explanation

The abstract provides the reader with a concise overview of the background information and progress toward the objectives. We recommend describing the main aspects of the research in the following order: **background - objectives - methods - implementation (results) - conclusions** - (optional: trial registration).

If applicable, include measurable KPIs and add keywords at the end of the abstract. In general, an abstract is around 150-300 words.

Answer:

Abstract:

Background: In interfacility transport care, matching ambulance response levels to patient needs is hindered by limited access to essential patient data during transport requests. Current systems struggle to integrate electronic health record (EHR) data due to privacy concerns and interoperability challenges, impacting timely decision-making and patient outcomes.

Objectives: This report details the implementation of Interfacility Transport Care (ITC)-InfoChain, a secure, blockchain-based platform designed to enhance real-time data sharing without compromising data privacy or EHR security.

Methods: ITC-InfoChain was implemented on AWS cloud infrastructure, employing Hyperledger Fabric as a permissioned blockchain. Key elements included participant registration, identity management, and patient data collection isolated from the sending hospital's EHR system. The client program submits encrypted patient data to a distributed ledger, accessible to the receiving facility's critical care unit at the time of transport request and EMS teams during transport through the PatienTrack web application. Performance was evaluated through KPIs such as data transaction times and scalability across transaction loads.

Results: ITC-InfoChain demonstrated strong performance and scalability. Data transaction times averaged 3.1 seconds for smaller volumes (1-20 transactions) and 6.4 seconds for 100 transactions. Optimized configurations improved processing times to 1.8-1.9 seconds for 400 transactions. These results confirm the platform's capacity to handle high transaction volumes, supporting timely, real-time data access for decision-making during transport requests and patient transfers.

Conclusions: The ITC-InfoChain platform addresses the challenge of matching appropriate transport units to patient needs by ensuring data privacy, integrity, and real-time data sharing, enhancing coordination of patient care. The platform's success suggests potential for regional pilots and broader adoption in secure healthcare systems. Stakeholder resistance due to blockchain unfamiliarity and data privacy concerns remains. Funding has been sought to support a pilot program to address these challenges through targeted education and engagement.

Introduction Items 3-5

Item 3: Context (M)

Describe the geographical areas, organizations, target populations, and implementation context. Consider social, cultural, economic, political, health care, and organizational barriers; infrastructures; and facilitators that may influence implementation elsewhere. Explicitly highlight whether a national digital health strategy exists and whether implementation is aligned with the strategy.

Describe the stage of the implementation (developing or adapting solution/piloting and evidence generation/package and advocacy/acceleration/deploying/scaling up/hand over or complete).

Examples

- "Concerto is a mobile application aimed at promoting empowerment for hospitalized patients. This implementation report will focus on the initial pilot study" [16]. "Switzerland has a digital health strategy, which has a component that focuses on the promotion of mobile health, based on the mhealth recommendations" [20].

- “This study was a pilot implementation of a chatbot-based mental health assessment performed in a real-world workplace setting, based on a cross-sectional analysis. The sample comprised employees of an industrial plant in Sao Paulo, Brazil” [18].
- “This study reports the user-centered design and feasibility study of a chatbot to collect linked data about diet, physical activity, weight, obesity risk, living area, and social network to support research regarding individuals and social causes of obesity and overweight. Here, we describe the user-centered approach applied in the design and development of the chatbot. We also present a pilot study to test the chatbot’s feasibility” [21].

Explanation

Context refers to the specific geographic, social, economic, and political factors that may influence the success of the implementation. These factors include geographic location, such as a specific region, country, or urban/rural setting, and the social and economic context, including demographics, income levels, and cultural norms that may explain user adoption. It is also important to understand whether there is a national digital health strategy in place and how the implementation fits into this overarching strategy, as failure to do so may result in missed opportunities for adoption. In addition, authors should mention the stage that the implementation report describes, as different reports may be issued for different stages.

Item 3: Context (M)

The ITC-InfoChain prototype is a blockchain-based solution designed to enhance decision-making in dispatching the most appropriate transport care units and qualified teams to sending hospitals, thereby mitigating potential adverse events during patient transport. The University of Maryland Medical System (UMMS), headquartered in Baltimore, operates within a complex regional network that includes collaborations with both UMMS and non-UMMS hospitals, and emergency medical services (EMS) such as the Baltimore City Fire Department. The ITC-InfoChain platform prototype was presented to stakeholders, including the head of the critical care unit at the University of Maryland Medical Center, the head of Baltimore city Fire department who acknowledged its potential to improve interfacility coordination. However, Stakeholder resistance due to blockchain unfamiliarity and data privacy concerns remains.

Social and Economic Context

UMMS is a private, not-for-profit healthcare system that generates revenue primarily from patient services and strategic partnership. While some government grants may be available for specific programs, UMMS largely operates without reliance on public funding. This economic landscape presents challenges to adopting new technologies like blockchain due to significant initial costs for infrastructure and ongoing maintenance expenses. Hence, utilizing an AWS-based infrastructure as presented in our prototype, can support the implementation of new technologies by providing scalable, secure, and cost-effective cloud solutions. AWS also facilitates rapid deployment and scalability, reducing reliance on extensive on-premises resources and thereby lowering overall operational costs.

Stage of Implementation

The ITC-InfoChain is at the prototype stage. While initial feedback has been positive, indicating potential as a scalable, secure solution for interfacility data sharing, further validation through a proposed pilot program is necessary to assess its real-world performance and address stakeholder concerns.

Item 4: Problem Statement (M)

Describe the health care or public health problem, challenge, or deficiency that the implementation aims to address. (If applicable, include a reference to the "health system challenge" of the WHO Classification of Digital Health Interventions [5] in the description.)

Examples

- "During the last decades, medicine has been moving from paternalistic approaches towards patient-centeredness and patient partnership and participation. Despite the high standard of healthcare in Switzerland, patients in hospital settings still face barriers to being fully involved in their own care and decision-making processes. This can lead to dissatisfaction with the care received, decreased trust in the healthcare system, and a lack of engagement in self-care and health management. Patient empowerment refers to a meta concept with no unique definition. It is however commonly accepted that empowered patients possess key capacities and resources to be able to participate in shared decision-making, manage their own health, and self-empower themselves. Patient empowerment can be associated with better outcomes, including mortality, especially for chronic diseases" [16].
- "Within hospital settings, a skills drills or emergency team may appoint a timekeeper in order to record clinical events in real time. However, within the home birth and other community-based settings, lone workers may also be faced with obstetric emergency, either alone or with little support. The unpredictability of such a scenario may be difficult to manage. As the need for clinical midwifery management becomes immediate, clinical record keeping may become retrospective and secondary to primary care" [22].

Explanation

The description of the health care or public health pain points, challenge, or deficiency that the intervention aims to address should be detailed and specific. This may include issues related to access to care, quality of care, patient outcomes, or health care delivery. It could also address public health challenges, such as disease prevention and control, health promotion, or population health management. It is important to clearly articulate the problems or deficiencies that the intervention aims to address to ensure that it is properly targeted and can effectively address the identified issues. Where appropriate, authors may wish to include a reference to the "health system challenge" of the WHO Classification of Digital Health Interventions in the description of this item [5].

Item 4: Problem Statement (M)

In interfacility transport care, a critical challenge is accurately matching ambulance response levels to the needs of patients, who may require specialized interventions en route to receiving hospital. This task is often complicated by limited access to critical patient data at the time of transport requests, hindering the ability to dispatch the most appropriate care unit and qualified transport team to the sending hospital. Currently, there is no efficient mechanism to import data from a sending hospital's electronic health record (EHR) into transport requests due to privacy concerns, interoperability limitations, and the sensitivity surrounding EHR data access. These constraints lead to delays and potential risks for critically ill patients awaiting transfer. Efforts to resolve this gap are hampered by disparate systems and a lack of reliable, secure methods to share real-time data between facilities and EMS teams at the time of transport request.

This challenge is aligned with multiple health system barriers as classified by the **WHO**, including **information-related issues** (e.g., limited access to reliable data, communication roadblocks) and **quality concerns** (e.g., inadequate integration of care and suboptimal workflow management).

Item 5: Similar Interventions (M)

Mention whether this implementation was inspired by another existing one. If so, what is the added value of your intervention, if any, compared to the initial one, and what, if anything, has been done differently?

Example

- "This health promotion program will be also based on successful and already tested health interventions for improving SRH of young people (including Cape Verdean youngsters), adapted to the needs and cultural-embedded values of the Cape Verdean young population, taking into account the perceptions of local health and educational professionals. This study presents an added value as a health promotion intervention with a comprehensive approach of well-being and quality of life across the life course. The framework of social prescribing and digital health literacy that supports this SRH intervention will allow social cohesion, throughout the cooperation between health and educational, from public or private sectors, including relevant stakeholders. A positive impact in social support perception is also expected, which is relevant from the perspective of sustainability. It is projected that the implementation and effectiveness assessment will embrace a pilot for national implementation, allowing to benefit several Cape Verdean's local communities, health services, educational programs, and policymakers" [23].

Explanation

References to similar interventions can provide links between different projects, acting as a catalyst for synergies and thus optimizing the impact of interventions on the target populations or within a particular health system. An intervention could build on another pilot as a foundation,

but be (1) an improvement, (2) a completely different initiative, or (3) a complement to the work of the base project. A critical point would be to demonstrate sufficient differentiation and strong added value of this specific intervention.

Item 5: Similar Interventions (M)

The ITC-InfoChain platform draws inspiration from recent blockchain solutions designed to improve data sharing in healthcare, such as MedRec, FHIRChain, Healthchain, and Action-EHR. Unlike these systems, which focus on public blockchain networks or centralized data models, ITC-InfoChain leverages HLF permissioned blockchain technology for secure, real-time data sharing between UMMS hospitals and EMS teams during interfacility transport. This approach offers significant added value by securely isolating critical patient data from the EHR system at the sending hospital, ensuring it can be accessed only by authorized providers throughout the patient's journey. ITC-InfoChain's unique use of data isolation and permissioned access enhances data security, efficiency, and compliance with privacy standards, addressing limitations seen in previous systems and focusing on critical care coordination.

Methods Items 6-14

Item 6: Aim and Objectives (M)

Describe the main objectives and the overall aim of the implementation. Describe how these will be measured using predefined primary and secondary outcome(s) and KPIs for this implementation and the expected intervention(s).

Examples

- "Our goal was to develop a system that will facilitate secure, trustable management, sharing, and aggregation of EHR data. Our patient-centric system allows patients to manage their own health records across multiple hospitals. The system will ensure patient privacy protection and guarantee security with respect to the requirements for health care data management, including the access control policy specified by the patient" [24].
- "Outcomes: Any positive or adverse health-based outcome and/or predictive indicators assessing pain and/or physical functioning/disability. Secondary outcomes: Any positive or adverse health-based outcome and/or predictive indicators assessing patient knowledge and understanding, self-efficacy, catastrophizing, and empowerment" [25].
- "The primary outcome was the change in insomnia symptom severity (measured by the Insomnia Severity Index) from baseline to postintervention. Secondary outcomes were sleep efficiency and nightly sleep duration (defined by sleep diary), global sleep quality (measured by the Pittsburgh Sleep Quality Index), depressive symptom severity (measured by the Edinburgh Postnatal Depression Scale), and anxiety symptom severity (measured by the Generalized Anxiety Disorder Scale-7)" [26].

Explanation

A detailed description of the objectives and the overall aim of the implementation gives the reader a clear idea of what the implementation is about. Authors should also clarify the difference between implementation objectives (the process; eg, with KPIs) and intervention objectives (the effectiveness), with outcome indicators, where appropriate. For example, authors might consider indicators or proxies that measure direct health outcomes (eg, hemoglobin A1C [HbA1c] for patients with diabetes), KPIs (eg, number of users, number of users properly trained, user satisfaction), and an indicator that assesses a particular process (eg, administrative time for patient admission). If no evaluation of objectives or outcomes has been carried out, it is important to explain the reasons for this decision.

Item 6: Aim and Objectives (M)

The primary aim of the ITC-InfoChain implementation was to enhance secure, real-time data sharing during interfacility patient transport in the region within the University of Maryland Medical System (UMMS) and its partner healthcare providers, addressing critical gaps in access to patient data and improving clinical decision-making at the time of transport request. ITC-InfoChain was designed to securely isolate and transmit key patient data to authorized personnel in receiving facilities and EMS transport teams, facilitating efficient dispatch of appropriately equipped transport units to prevent adverse events during patient transfer, especially for patients in critical conditions.

Objectives:

The system's effectiveness was assessed by measuring transaction completion times and data access latency (see Appendix B and the result section of the manuscript). Presentations to stakeholders, including the head of the critical care unit at the University of Maryland Medical Center, highlighted its potential for enhancing coordination at the time of transport requests (support letter, available upon request).

Ensuring data privacy and security was a core objective in designing the ITC-InfoChain platform architecture before implementation. The platform employed a secure, isolated data exchange process by extracting patient data from the sending hospital's EHR and storing it in a disconnected Data Exchange (DX) file. The client program reads this data and submits encrypted transactions to a permissioned blockchain ledger, providing real-time access to EMS and receiving staff via the PatienTrack application at the time of transport requests and during transport to the receiving hospital. Additional security measures included strong identity management, data encryption, automated data sharing through smart contracts, and enforced access controls to protect privacy. Finally, the project aimed to demonstrate the feasibility of the platform prototype without compromising data security and privacy, ensuring appropriate interoperability at the time of data collection and extraction in the sending facility.

Item 7: Blueprint Summary (M)

Describe the design and key features of the intervention and key points of the implementation strategy and roadmap.

Examples

- "It (Concerto) was conceptualized and prototyped during a hackathon in 2015 by a multi-disciplinary team including healthcare and IT professionals as well as one patient. Building on the hackathon prototype and after a feasibility study, the Geneva University Hospitals launched a project aiming at developing a fully functional mobile application to be deployed on institution-owned iPads in 4 divisions: oncology, neurorehabilitation, orthopaedics, and paediatrics, and assessing its effectiveness. Following this pilot study, the mobile application was further refined and deployed institution-wide in a Bring-Your-Own-Device (BYOD) approach" [16].
- "…we developed pain SELfMAnagement (SELMA) as a text-based health care chatbot (TBHC) for the self-management of chronic pain. A TBHC is a conversational agent that supports health professionals in the delivery of evidence-based interventions in a ubiquitous and fully automated fashion with simple text-based messages and media objects (eg, videos, podcasts). A TBHC aims to deliver the treatment and to increase working alliance by communicating therapeutic goals and tasks in an empathetic way. Against this background, we here describe the design and implementation of SELMA, an 8-week smartphone-based TBHC intervention for self-management of pain by patients with ongoing or cyclic pain, and present findings from a pilot randomized controlled trial, in which effectiveness, acceptance, and adherence were evaluated" [27].

Explanation

When describing the implementation strategy and the main features of the intervention, authors should bear in mind that intervention refers to the deliberate and intentional effort to change a situation or behavior and implementation refers to the process of achieving the goal (how the intervention is carried out). Outline the implementation strategy with key milestones. This will provide a more complete understanding of the implementation process and any deviations from the original plan. If possible, authors can include "Digital Health Interventions" and "System Categories" from the WHO Classification of Digital Health Interventions [5].

Item 7: Blueprint Summary (M)

The design and key features of the intervention:

The ITC-InfoChain AWS-hosted platform simulates a connection between UMMS hospitals and EMS transport teams, ensuring that critical patient information is accessible at the time of transport request and during transport, facilitating efficient dispatch of appropriately equipped transport units with transport teams, all without directly accessing the sending facility's EHR.

key Features of design:

Key features include (1) a data exchange (DX) file that securely isolates patient data collection from the sending facility's EHR at the transport request stage, by collecting data and disconnecting from it to maintain privacy; (2) a client program reads data from the DX file and connects to the permissioned blockchain network to submit TXs.; (3) a Hyperledger Fabric network providing peer to peer connection among hospitals serving patient data access at the time of transport request and during transportations. It ensures strong member identity

management, certification issuance, and data encryption for secure patient data sharing; (4) smart contracts for automated, compliant data sharing; and (5) PatienTrack, a web application enabling EMS and receiving hospital staff to view patient data in transport request time and during transfer.

key implementation strategy

The ITC-InfoChain platform is strategically designed to ensure secure, real-time patient data sharing through an isolated data exchange process. At the transport request stage, the sending hospital's EHR data is extracted and securely stored in a Data Exchange (DX) file, which remains disconnected from the EHR to uphold privacy. reads data from the DX file and connects to the permissioned blockchain network to submit TXs to be recorded on a permissioned blockchain ledger. This setup enables real-time access for EMS and receiving hospital staff via the PatienTrack application, ensuring compliance with identity management and data encryption protocols. This strategy not only supports seamless interoperability between disparate systems but also automates data sharing through smart contracts, which enforce access control and privacy.

Roadmap:

Following initial testing, the next phase includes a pilot program—pending grant funding and UMMS support—to test ITC-InfoChain with anonymized patient data in real-time transport scenarios. After pilot validation, the roadmap envisions evaluating the key stakeholders perspectives in an empirical study.

Item 8: Technical

Design (M)

Specify reasons for developing or choosing this tool. Does it combine several tools? Provide a brief description of the tools (functionality and architecture) and how it fits into the health enterprise architecture and investment roadmap (if applicable). Indicate whether the solution is based on an existing solution or has been developed or purchased specifically for this intervention.

Describe the type of technology used (eg, artificial intelligence [AI] applications) and the license of the technology (open source, free, commercial, intellectual property [IP] ownership, etc) and include code documentation (if available), a link to the application, and a link to wiki or the project website.

Examples

- "The Jamii ni Afya mobile app is built on the Community Health Toolkit (CHT), an open source global goods platform developed to support community health workers globally. This platform was selected by the Zanzibar government due to the following reasons: it is open-source and uses well-known components and frameworks; it has a growing community that can be leveraged for support; it can be hosted in a local datacenter; the

skills required to configure health worker tools are found among Ministry ICT staff and easily available in the local market. In addition, CHT runs on low-end Android smartphones and has offline functionality, which is critical in Zanzibar where network connectivity is not guaranteed"[15].

- "The application was developed in a web-based, responsive, coding language, encapsulated for iOS and deployed on institution-owned iPads using a mobile device management. The key arguments for internal development over acquisition of a commercial solution were that (1) most of the development work was about interfacing with the Hospital Health Information System, (2) no mature commercial solution was available at that time, to our knowledge" [16].
- "The Java programming language was used to develop a native app focusing on the Android platform, in which personal and medical data are maintained using the SQLite database. The first version of the app was con-structed and presented to the same nephrologist still in 2015. This aimed to make sure that all specified requirements were incorporated into the app" [28].

Explanation

Technical design explains the reasoning and process behind the implementation of a specific functionality. Understanding the lessons learned or failures (assessing the actual technical design against the original intent) can help improve digital health development standards and address common technical issues encountered in implementations.

Item 8: Technical

Design (M)

Based on the system Architecture provided in Figure 1 in the manuscript, the following functions are performed by each component and the reason behind the tool selection.

Function 1: **Collecting patient data from sending facility's EHR at the time of transport Request in a secure manner:** once a transport request is initiated by the sending hospital, a pre-configured job in the hospital's database extracts the necessary patient data from its EHR system and securely stores it in a file (data exchange file – DX) on the hospital's file system, which serves as an interoperability layer between the EHR and ITC-InfoChain.
Data collection isolation: Once the data is successfully collected, the file is disconnected from the EHR, ensuring separation of data collection from data extraction and recording on the distributed ledger.

Function 2: Extracting patient data from DX file at the time of transport Request: The **Client program** extracts data from the DX file and encrypts it.

Function 3: **Submitting the data recording request (Transaction or TX) to the Hyperledger Fabric**, a permissioned blockchain network**: The client program** submits the TX to the network.

Function 4: Recording patient data on the distributed ledger on the HLF network: **A smart contract**, known as "chaincode" in Hyperledger Fabric (HLF) with other network components during mutual connections records data in the ledger.

Function 5: Access patient critical data in the receiving facility's critical care Unit's patient portal to assess patient condition acuity and dispatch an appropriate ambulance and qualified transport team to the requesting hospital (the core aim of this implementation):

Tools selection for the prototype:

Technologies used for the implementation

As a peer-to-peer network directly connecting hospitals involving in Interfacility Transport Care, we selected Hyperledger Fabric, a permissioned blockchain technology. Hyperledger Fabric was chosen due to its strong membership and identity management, certification issuance, and data encryption capabilities, which align with healthcare privacy standards. We created a subnetwork of HLF network also known as channel which comprised of three members: a sending hospital, a receiving hospital, and an EMS ITC provider. Other channels can be created in case any hospital intends to share some data mutually and confidentially or run another function on the network.

We developed the client program in Python, chosen for its cross-platform compatibility and strong community support, which make it adaptable and easy to deploy across different systems. The PatienTrack web application was also built in Python using the Django framework.

A smart contract, known as "chaincode" in Hyperledger Fabric (HLF), was developed using the Go language, which is optimized for HLF. Go was chosen for its performance, efficient memory management, and ability to handle multiple concurrent requests. Additionally, it benefits from strong community support. Other languages are also compatible for chaincode development.

Links to Codes

The following components are integral to the system:

- **Client program**: This application retrieves patient data from a CSV file, creates a transaction (TX), and sends the request to the chaincode, a smart contract on the Hyperledger Fabric (HLF) network.

  https://drive.google.com/drive/folders/13ntoVaSO5eDoWlodtlJGn2q08CgF3EXK?usp=sharing

- **Chaincode ("patientdirectory.go")**: This smart contract records transactions and patient data on the distributed ledger, which includes both the blockchain and the state database, following a consensus mechanism that ensures data integrity across the network.

  https://drive.google.com/file/d/1A3DaxwLdAfOZAFU-xZa-K7fGBNdCW96o/view?usp=sharing

- **PatienTrack Web App**: This application connects to the HLF network to read patient data from the ledger, displaying it in the patient portal at the receiving facility when a transport request is initiated. The app also allows EMS personnel to view and add data during the patient's transport to the receiving hospital.

  https://drive.google.com/drive/folders/1C0TQPH828ViPW0_b6P0nJLKCm7cTelGr?usp=sharing

Item 9: Target (M)

The target refers to the focus or recipient of the intervention. It is the individual, group, system, or problem that the intervention aims to change or improve. Describe the characteristics of the targeted "site(s)" (locations, staff, resources, etc) for implementation and any eligibility criteria, as well as the population targeted by the intervention and any eligibility criteria.

Examples

- "Concerto is a mobile application designed to promote empowerment for patients still hospitalized or released from the hospital and ease the support provided by their caregivers, in Switzerland" [16].
- "This app development is conducted as it is essential for fulfilling the need to increase midwives' competencies integrated with their services based on mobile health that can facilitate midwives to develop themselves according to their profession. '' subjects included midwife participants in West Java Province, Indonesia" [29].
- "Jamii ni Afya leverages government guidelines and global best practices to guide CHWs using digital technology in delivering high quality, health education and counseling services in maternal and child health, nutrition, water, sanitation and hygiene (WASH) and early childhood development" [15].

Explanation

The targets may include the sites, the staff, or other resources needed for the implementation, as considered by the authors.

It is important to accurately describe the targets of the intervention, including those added during the implementation process, in order to provide a full understanding of the scope and objectives of the intervention. In some cases, the rationale for the choice of targets may be inadequately explained or overlooked, as the authors may assume that it is obvious from the context. It is important to clearly describe the eligibility criteria for all targets of the intervention to provide a thorough understanding of the target selection process. An exhaustive description will facilitate not only the assessment of relevance but also, where appropriate, the replication of the intervention by others.

Item 9: Target (M)

The ITC-InfoChain platform targets critical interfacility patient transport coordination within the University of Maryland Medical System (UMMS), involving EMS teams and healthcare staff responsible for dispatch and receiving patient data during transfers. The primary intervention sites are UMMS hospitals participating in interfacility transport, along with EMS teams who are authorized to access and update patient data in transit. Eligibility for implementation includes healthcare facilities within UMMS that handle critical care cases and rely on efficient, secure data sharing during patient transfer.

The platform's intended users are hospital staff in critical care units, transport dispatchers, and EMS team members. These users are essential to ensuring timely, well-coordinated care for patients requiring interfacility transport, especially those at high risk. In the simulated phase, ITC-InfoChain uses mock data to test data-sharing workflows and system efficiency across authorized UMMS and EMS personnel. If granted approval and funding, the pilot phase will utilize anonymized patient data in a real-time environment, focusing on UMMS facilities where critical patient transport is most frequent, and ensuring data privacy and security remain paramount throughout the implementation.

Item 10: Data (M)

Describe the data governance, including the life cycle (collection, processing, storage, modification, sharing, suppression); data ownership (mention whether patients actually have access to the data); data protection measures; confidential use of routine data; expected level of data integration; data for research; cross-border data agreement, if any; the applicable legal framework; and how the project complies with it. Describe data consent: Has patient consent been obtained? Describe the approach to data protection and cybersecurity (eg, security by design, privacy by design) and where the data are hosted (eg, in-country, cloud-based, hybrid model). If applicable, describe the government's data policy.

Examples

- "Concerto was mainly 'read-only' for personal health data present in the HIS and for unsensitive, unpersonal information. The information patients accessed from the HIS were part of its medical record. According to the Swiss law, every patient owns data from his medical record, except for personal notes of healthcare professionals which were out of the scope of concerto. Accordingly, concerto facilitated the access to data already owned by the patients. The only personal information entered in concerto was questions patients may have before interaction with caregivers. This information was stored locally on the iPad. As iPads were erased and reinitialised between patients, this information was systematically deleted. Secure login information based on the patient ID number and an SMS challenge also protected from unwanted access to sensitive personal information. Overall, the project was compliant with the Swiss law for data protection" [16].
- "The program consists of a clinic computer that hosts the local database of patient names and their phone numbers. A secure server, hosted by Sawubona Health, synchronizes with the local database. Each week, the server uses PHP scripts (commonly used for web development) to automatically generate the messages and pass them and the

corresponding phone numbers to a BulkSMS service provider (Celerity Systems LTD, South Africa) for distribution" [30].

Explanation

Effective data strategies are essential for the successful implementation of digital health, while complying with legal frameworks on data ownership and privacy. Transparency of data used for decision-making is critical to facilitate evaluation of interventions. Ethics committees may require reporting of the detailed data strategy.

Item 10: Data (M)

The HLF network in ITC-InfoChain enables direct, secure data sharing between hospitals and EMS, bypassing the need for third-party data management. Traditional systems require hospitals and EMS to send patient data to a third-party database due to interoperability and privacy issues, which then distributes it to others, resulting in delays and loss of direct data ownership. ITC-InfoChain's peer-to-peer structure retains data ownership within hospital nodes, ensuring instant access to patient data during transport. Unlike centralized systems, which are prone to security breaches, data privacy risks, and single points of failure, the permissioned blockchain framework in ITC-InfoChain provides a more secure, governed solution.

Data Lifecycle and Security:
The data lifecycle involves data collection, data extraction and recording in the distributed ledger on HLF network, and real-time access at the time of transport request and during transport. Data modification is not allowed by receiving facility's staff and during transport, only EMS can add data to the existing ones en route to be visible to authorized members. All data is hosted on AWS's U.S.-based cloud infrastructure, complying with in-country data requirements.

Ownership, Privacy, and Compliance:
Operating under HIPAA and UMMS's data governance policies, ITC-InfoChain ensures that data ownership remains with the originating hospital, while transparency is achieved through a shared, permissioned ledger accessible only to authorized stakeholders. Although patients do not directly access the system during transport, consent protocols are managed at the hospital level. Data is fully encrypted, with strict access controls to maintain confidentiality and data integrity across the transport process.

Item 11: Interoperability (M)

Describe the interfaces (what other systems does the tool connect to) and the standards that were used (which specific ones and rationale of choice; eg, semantic ontologies, such as the International Classification of Diseases [ICD], Systemized Nomenclature of Medicine – Clinical Terms [SNOMED CT], Logical Observation Identifiers, Names and Codes [LOINC], or technical standards, such as Health Level Seven Fast Healthcare Interoperability Resources [HL7 FHIR]).

Examples

- "The initial version of the application connected directly to the custom-made Geneva University Hospitals Health Information System (HIS) using proprietary interfaces. Further versions of the application have used industry standards such HL7/FHIR to connect to REST/APIs on the hospital side. This new architecture has required significant evolutions both on the application and the HIS side" [16].
- "NextGen Connect Integration Engine is also a cross-platform engine allowing the bidirectional sending of messages in many supported standards (eg, HL7 V2, HL7 V3, HL7 Fast Healthcare Interoperability Resources, DICOM) between systems and applications. Semantic interoperability provides interoperability at the highest level, which is the ability of two or more systems or elements to exchange information and to use the information that has been exchanged" [31].

Explanation

At the national level, the implementation of harmonized interoperable systems is essential to ensure sustainability and cost-effectiveness, as well as to optimize coordination between stakeholders. Interoperability can be achieved at different levels (technical, syntactic, semantic, organizational, or even legal). It is recommended to fully describe all standards and to mention whether interoperability policies have been applied (in an organizational or national context).

Item 11: Interoperability (M)

The following workflow design and implementation, thoroughly describes how the ITC-InfoChain platform interfaces with the sending facility's EHR via the Data Exchange File (DX), as well as how it connects with the blockchain network using Hyperledger Fabric, SDK APIs, and proprietary APIs.

The ITC-InfoChain platform is designed to use a Hyperledger Fabric connection profile, SDK APIs, proprietary APIs, and data files to facilitate interoperability as follows. We employed DX File to enable data collection and interoperability between the sending facility's EHR and the blockchain network. The DXF file collects data from the sending facility's EHR, disconnects, and then supports subsequent data retrieval and recording on the Hyperledger Fabric (HLF) network. This setup ensures that the hospital health information system remains disconnected from the blockchain network during data extraction and recording.

When a transport request is initiated, a job setting in the sending facility's database system triggers a connection to the EHR, collects relevant patient data, and stores it in DX file before disconnecting from the EHR. The client application then connects to this file, automatically extracting and encrypting the data before submitting it to the peer node on the HLF network for recording. This workflow allows ITC-InfoChain to securely manage data without requiring a direct EHR connection, addressing interoperability barriers through a disconnected data exchange model. The client application uses a network profile to connect to the network, linking participating hospitals. Additionally, a web application called PatieTrack utilizes SDK APIs to

connect to the blockchain network to retrieve and display data to any authorized entities as members of network.

Currently, the data shared at the time of transport request is recorded on the permissioned blockchain is not recorded in the receiving facility's EHR, so data standards like HL7 FHIR or SNOMED CT have not been employed. However, the platform is designed to support future integration of standards, which could enable secure, real-time API-based data transfer directly into receiving facility EHRs. Future implementations of these standards would facilitate compatibility across a broader range of hospital systems and enhance data interoperability at national and organizational levels, enabling a more seamless data-sharing process and automated information transfer across healthcare systems.

Item 12: Participating Entities (M)

Describe the following:

Implementing organization(s): type of organization(s), mission, leadership, vision, etc.

Government involvement: Describe whether the government was involved in the implementation, at what level, and at what stage(s).

Partners: Describe all partners (organizations) and their role in the implementation.

Funders: List all actors and stakeholders who have funded or invested in the development of the implementation (if different from the implementation, eg, using an existing digital health intervention). Indicate their level of involvement in terms of funding.

Mention which entity will own the final product and intellectual property after the implementation phase.

Examples

- "mTrac is a government initiative that originated as a pilot project within a Millennium Villages Project and Foundation for Innovative New Diagnostics (FIND). It was then handed over to the Government of Uganda for launch and scale up in December 2011. The Ministry of Health (MoH) fully owns and operates mTrac and it began to roll it out in four phases, each covering approximately twenty-eight districts.With financial support primarily from the UK Department for International Development (DFiD), this is done in three key ways. Firstly, via SMS, in order to transmit weekly surveillance reports (i.e. information on disease outbreaks and stocks of anti-malarials) from health facilities to the MoH and District Health Offices (DHOs). The MoH receives technical support from UNICEF and WHO, as well as financing from DfID, but mTrac is formally governed via a government-led Steering Committee chaired by the National Medical Stores, as well as via a dedicated eHealth Technical Working Group (TWG)" [32].
- "The feasibility study and initial concept were self-funded by the eHealth and Telemedicine Division of the Geneva University Hospitals and included salaries for a junior developer and a senior project manager. The project pilot was then funded by the

Fondation Privée des HUG and included the aforementioned salaries as well as necessary materials (in particular iPads, covers and software licenses). No direct state-funding was provided at that stage of the project" [16].

Explanation

Identify all the different stakeholders involved in implementation. Partnerships can be key to successfully initiating and scaling up digital health tools. Therefore, understanding the respective roles of each participating entity will facilitate a clear understanding of the strategy and interactions.

Item 12: Participating Entities (M)

The ITC-InfoChain platform was conceptualized and designed by the authors, with the prototype developed and implemented on AWS cloud infrastructure. This work addresses a problem identified by the University of Maryland Medical Center's critical care unit and Dr. Ben Lawner, Medical Director of the Baltimore City Fire Department (support letter, available upon request).

Item 13: Budget Planning (M)

Describe the planned PKI

budget for implementation (include costs such as change management, user training, project management, technology pricing, total cost of ownership). If possible, include actual costs, otherwise describe the range or percentage of the total budget. Indicate the time frame covered by the budget. Describe the budget for the intervention (eg, development, purchase or adaptation of a free tool); if possible, include actual costs, otherwise describe them as a percentage of the total budget.

Examples

- "The cost of Corrie was estimated to be $229 per month per patient for a 1-year use term ($2750 per year). Based on this cost estimate, the use of the DHI leads to a cost-savings of $7274 per patient compared with standard of care alone (ie, $10,024 − $2750 = $7274). The $2750 figure is composed of a: (1) Bluetooth blood pressure monitor (~$40); (2) refurbished smartwatch (~$250); (3) medication pillbox (~$10); (4) tote bag (~$12.50); (5) printed instructions (~$5); and (6) clinical support for onboarding and maintenance of platform by engineering inclusive of server storage fees and helpline access per user (~$2432 annually)" [33].
- "This project was estimated at CHF 60, 000: CHF 8,000 for the adaptation of the Mediboard software; CHF 33,000 for investments (intranet network, computer equipment - servers, computers, switches, etc.), communication and marketing of the project; CHF 14,000 for operations (project management, change management, management of steering and working meetings - with Maternity user group); CHF 2,000 for training and

support of users; and finally CHF 3,000 for the evaluation of the solution by an external evaluator of project" [17].

Explanation

A comprehensive budget plan with detailed cost estimates for both the implementation and the intervention is important for conducting future economic evaluations and for estimating future funding needs; budget planning helps to optimize resource allocation decisions, which can influence the effectiveness of digital interventions. One dimension of long-term ownership that should be considered is whether the tool has a "technical owner" who can provide technical support and own the functionality of the tool in the long term before it is adopted [34]. The costs associated with such resources need to be properly assessed. Where possible, authors should include real costs.

Item 13: Budget Planning (M)

The development and implementation of the ITC-InfoChain platform were completed over a two-year period using self-funding. The ongoing maintenance on AWS cloud infrastructure incurs a monthly cost of over $40, with a billing snapshot provided as supplementary documentation. Following development, Morgan State University assumed ownership of the prototype and covers these maintenance expenses.

In addition, the platform's blockchain network was upgraded from HLF version 2.2 to version 2.5, funded through an $8,000 internal grant from the Office of Technology Transfer at Morgan State University. This upgrade enhances performance and security, supporting ITC-InfoChain's long-term scalability.

Item 14: Sustainability (M)

Describe the business model, including the sustainability model (financial, environmental, etc). If possible, relate outcomes to costs to assess sustainability. Describe long-term exit strategies and all dimensions considered to sustain the project after the end of the funding period. If applicable, describe the potential institutionalization of the project.

Examples

- "The University of Pittsburgh team provided all capital expenditures for the wellness center's construction, as well as telehealth equipment, personnel recruiting and training, power and water infrastructure, and operating expenses for the first two years. The model was designed to be self-sustaining after two years of operation by 1) reimbursing services through government programs such as health insurance and 2) returns from the agribusiness enterprise that provide income and employment to the local community and generate enough economic value to support the Tuver Project. At the end of Year 1, all healthcare services, including specialty and super-specialty teleconsultations and basic laboratory tests at the wellness center were available at no cost. The project provided medicines free of charge to those with incomes below the

poverty line and at discounted rates to all other patients. The program offered outreach services and menstrual hygiene kits at no cost to community members. The Common Services Center provided services at nominal prices predetermined by the relevant governmental authority" [35].

- "To ensure the sustainability of this project, several actions have been taken. These include: the alignment of the project's objectives with those of YCH and its implementation with the agreement of the Director of YCH; the commitment of the Director to fund one third of the budget; the establishment of a steering committee including all YCH stakeholders, the integration of the YCH Information Technologies manager as deputy project manager, the implementation of a user group to assist the project group in the design and the implementation of the project, the designation of local champions (in the maternity) to provide leadership in the use of the CIS and finally, the official launch (supported by a document signed by the Director) of the beginning of the use of this CIS in the maternity" [17].

Explanation

Sustainable financing is critical to scaling up digital health interventions, but it is also often perceived as 1 of the most challenging parts of the process. Understanding whether the benefits of digital health services are balanced with their impact on the planet is important to ensure the continued advancement of the technology, while promoting sustainability. Authors should explain the sustainability plan (financial, environmental, etc) for implementation. If applicable, describe long-term exit strategies and all dimensions considered to sustain the project after funding ends.

One approach recommended by National Health Service (NHS) England to make digital health solutions sustainable in health systems was "to reject the traditional linear model of the innovation process in favour of an interactive model where implementation is not an afterthought but a primary focus of co-design efforts" and to move away from a focus on the technology itself to a focus on how digital technologies will be integrated and used in services in order to understand the context, environment, and constraints of the people who will use them and the target populations who will benefit from them [36].

Item 14: Sustainability (M)

The ITC-InfoChain platform's sustainability model is underpinned in several strategic actions designed to ensure its longevity and adaptability in the interfacility transport sector. Developed and implemented with self-funding, the platform now incurs low ongoing maintenance costs on AWS, approximately $40 per month, which are covered by Morgan State University. To enhance the network's functionality and security, the system was upgraded from HLF version 2.2 to 2.5 with support from an $8,000 grant provided by Morgan State University's Office of Technology Transfer.

The long-term sustainability of ITC-InfoChain is based on a financially self-sustaining model, with Morgan State University maintaining technical ownership. This arrangement secures access to necessary resources, technical support, and ongoing development. In the future, the ITC-InfoChain platform could be supported through grant funding or partnerships with healthcare organizations such as UMMS, allowing it to expand to additional hospitals or EMS services in need of secure transport data solutions. Further institutional support from Morgan State University ensures that the system can be adapted to meet emerging healthcare data needs, enhancing its scalability and potential integration with other healthcare networks.

If future funding or partnerships are unavailable, Morgan State University will serve as the platform's long-term steward, ensuring continued operational capacity. The modular, cloud-based design of ITC-InfoChain also reduces environmental impact, as AWS infrastructure allows for resource scaling as demand changes, minimizing excess energy use. With ongoing institutional backing and low maintenance requirements, ITC-InfoChain is positioned for sustainable, scalable integration into healthcare transport networks.

Implementation (Results) Items 15-18

Item 15: Coverage (M)

Describe whether the coverage of implementation is international, national, regional, or at the level of, for example, municipalities. If coverage is subnational, describe the regions. Provide information about the relative importance of the coverage (eg, percentage of the eligible population covered).

Examples

- "The Geneva University Hospitals, a 2000-beds Swiss teaching hospital, launched a project to develop a fully functional mobile application to be deployed on institution-owned iPads in 4 divisions: oncology, neurorehabilitation, orthopaedics, and paediatrics. Following this pilot study, the mobile application was refined and deployed institution-wide following a Bring-Your-Own-Device (BYOD) approach. Concerto was then further extended to encompass the ambulatory setting and facilitate the transition from hospital care to home care" [16].
- "This study took place at Webuye County Hospital, a rural hospital in Bungoma County with a catchment population of 500,000 people. It is estimated that approximately 44% of the population are children, 15 years. The hospital TB clinic cares for 200 patients with active TB annually. More than 1500 children are seen monthly in the pediatric outpatient clinics (nutrition, maternal child health, acute care)" [37].

Explanation

When describing the coverage of the implementation, authors might consider comparing the planned coverage (both geographical locations and population) with the actual results. Any discrepancies observed should be mentioned, as well as suggestions for better understanding the reasons for the differences.

Item 15: Coverage (M)

The ITC-InfoChain prototype, created on AWS, simulates data sharing at the time of transport request and during transport, with plans for an initial pilot at the regional level involving 11 hospitals affiliated with UMMS and several private and public emergency medical agencies engaged in interfacility transport care.

Item 16: Outcomes (M)

Describe the primary and other outcomes of the implementation. Detail the actual outcomes, using the predefined outcome measures (if applicable).

Example

- "The FORA device, when used as an intervention within standard care as a control, had a moderate to large between-group effect on medication adherence 1 month posttest (d=0.77), 2 month posttest (d=0.88), and continued to stay just as effective, if not become even more so, at the 3-month follow-up (d=1.02)" [38].

Explanation

Authors should comprehensively describe the different levels of outcomes of the implementation and then evaluate the achievement of these against the initial strategy. This will help better assess the effectiveness of the project.

Item 16: Outcomes (M)

The ITC-InfoChain prototype was successfully implemented on AWS cloud infrastructure in a simulated environment to evaluate its data-sharing capabilities in interfacility transport scenarios. Performance testing revealed that data transaction times were consistently fast across various patient volumes. Specifically, data recording speeds averaged 3.1 seconds for volumes of 1, 5, and 20 patient entries and approximately 6.4 seconds for 100 patient entries, with optimized configurations achieving 1.8 to 1.9 seconds for 400 patient entries. This rapid data recording met the predefined performance target, ensuring timely availability of patient data for authorized personnel at receiving facilities. Additionally, data access latency remained under 3.1 seconds, allowing EMS and hospital staff prompt access to relevant information at the time of request and during patient transport.

Detailed performance metrics under different configuration settings are presented in Tables 1–5, which summarize the transaction processing times observed across various batch sizes and message counts. These tables illustrate the system's scalability and efficiency, with optimized configurations enabling rapid processing of high transaction volumes, making the ITC-InfoChain suitable for high-demand, data-intensive scenarios in interfacility patient transport (see Appendix B). These findings are supported by images captured during data retrieval and recording while the network, client app and patienTrack app was running on AWS cloud infrastructure (Appendix C, available upon request).

Item 17: Lessons Learned (M)

Describe any lessons learned from the implementation experience that could be used to improve future outcomes. This could include, but is not limited to, success factors, implementation challenges, or budget considerations.

Success factors: Describe factors that positively influenced the implementation (eg, involvement of key stakeholders). In addition, describe contextual factors that may have positively influenced the results (eg, new legislation that facilitated adoption).

Challenges to implementation: Describe challenges (process related, such as resistance to change, but also technical). Include contextual factors that may have affected the achievement of outcomes, such as an unexpected change of government or “opposing key players” who, despite potential participation, may hinder implementation (eg, software companies managing regional digital health may act as barriers to innovation).

Budget: Describe whether the implementation budget was met, and if not, why not. In addition, detail the expected operational costs (eg, licensing, maintenance, human resources, updates to in-house developments) to estimate the total cost of ownership. Include actual costs, otherwise describe them as a percentage of the total budget.

What recommendations can be drawn from the lessons learned?

Examples

- “Lessons learned, presented in the results section, are summarized in Table 1. The generalisability of our findings is low as they constitute the report of one implementation and may obviously vary in a different implementation context, such as another category of hospital, another healthcare system or another cultural context. ‘Overall, the order of magnitude of the project costs was comprised between 150k and 200k CHF, from which 25% was used for materials’” [16].
- “Related to the implementation of the NAA in practice, some unforeseen challenges arose that needed to be solved. For example, three healthcare workers were not able to attend the 2-day NAA training and were therefore trained individually at their healthcare facility. Also, of the in total 15 tablets five broke down, of which three could not be replaced and some remaining ‘bugs’ in the application were discovered. Healthcare workers did not always express their true opinions or would not call the technical support team when problems occurred. There was no script available in the implementation plan to guide responses to unexpected changes making it difficult to solve problems and ensure sustained use of the programme. Based on the lessons learned during the process of developing and implementing the NAA we recommend future programme developers to (1) engage the community and listen to their insights, (2), focus on clear programme goals and the desired change, (3), consult or involve a behaviour change specialist, and (4), anticipate potential problems in unexpected circumstances” [39].

Table 1. Main lessons learned and associated perceived relevance [16].

Lessons learned
Minimize or, if possible reduce the workload of caregivers.
Plan protected time to train end-users.
Communicate regularly to keep caregivers engaged.
Select convinced and influential superuser.
Wait for a killer function to implement the application.
Maturity of HIS in terms of interoperability standards facilitates implementation

Explanation

Failure to evaluate the different stages of development may prevent the understanding of key lessons that could support greater effectiveness and adoption of these initiatives. Authors should aim to formulate any recommendations that could serve as guidance for more effective implementation of digital health tools/systems.

Item 17: Lessons Learned (M)

The implementation of the ITC-InfoChain prototype within a simulated environment offered valuable insights into the factors influencing both the potential and challenges for piloting the platform.

Success Factors:
Key stakeholders, including the head of the Critical Care Unit at the University of Maryland Medical Center, showed strong support, recognizing ITC-InfoChain's potential to improve data sharing during critical patient transports. This endorsement, documented in a supplementary letter, provided credibility to the project and helped to build interest in further evaluation. Additionally, the AWS cloud infrastructure's scalability and cost-efficiency enabled efficient data management, simulating the benefits of a dedicated, secure hospital data center environment. These strengths underscore the importance of stakeholder engagement and flexible infrastructure in advancing digital health solutions.

Challenges to Implementation:
Resistance from some stakeholders within UMMS posed a challenge, primarily due to concerns about data privacy and limited familiarity with emerging blockchain technology. Even though ITC-InfoChain was proposed to handle pseudonymous patient data only, the fear of data exposure persisted, indicating the need for a comprehensive education component to improve understanding and acceptance of the technology. Additionally, uncertainty around the total cost of full implementation and maintenance, despite AWS's "pay-as-you-use" model, highlighted the

importance of a clear, predictable budget. Stakeholders also expressed concerns about the need for exact cost estimates for long-term maintenance and operational requirements, especially in a health environment sensitive to budget fluctuations.

Item 18: Unintended Consequences (NM)

Describe any unintended consequences (positive or negative), harms, or negative side effects (if any).

Examples

- “Our strategy to use institution-owned iPads has brought important additional workload on care teams as they were in charge to manage iPad fleets in their division. There was also positive consequence for Concerto which was able to be exposed in the Information systems” [16].
- “During this project and after implementing some approaches for change management (setting up a user group, identifying local champions, training of users, implementing news processes based on the procedure manual, setting up user support), we experienced an unintended or unexpected event which was staff reluctance to use the system after implementation due to lack of financial motivation. This situation required the project management team to put in place a special documentation gratification (per diem introduced to encourage staff to enter patient information into the system)” [17].

Explanation

During the implementation and intervention phases, unexpected events may occur that could potentially hinder implementation or, on the contrary, increase its impact. It is therefore important to identify any unintended consequences. This important assessment can lead to the revision or creation of standards.

Item 19: Discussion (M)

Provide a summary of the conclusions and future implications.

Examples

- “As in reaction to one of the main lessons learned, a Bring-Your-Own-Device version of the application was developed. With this version, every patient was able to use the application on its personal device (computer, tablet, or smartphone). This was done to limit the workload on caregivers and improve the adoption rate. New functionalities, including the possibility for patients to choose his meal, were also developed to answer unmet needs for both end-users and stakeholders impacted by the implementation of the application (i.e.: caregivers). A dedicated implementation report describing this project phase shall be submitted soon” [16].
- “The results from this study are added evidence that chronic disease risk reduction is achievable through a variety of modalities, including digital-based programs with human coaching. With the added advantage of accessibility and scalability, digital programs with

human coaching should be an important part of the comprehensive health improvement solution for chronic disease risk reduction for older adults. This study demonstrated that older adults who agreed to participate in this program were able to engage meaningfully and gain important health and wellness benefits during a relatively short time frame" [40].

Explanation

Authors should use this summary section to help readers understand the main conclusions of the report. This section should also help the evaluators quickly understand the stakes and various future implications of the implementation.

Item 19: Discussion (M)

Limitations:

This study has several limitations: First, communication and computational costs could not be accurately measured, as a long-term, fully operational version of the solution is required to assess these factors over an extended period. Second, this prototype was tested in a simulated environment, limiting the ability to observe real-world system responses, including data latency under varying network conditions across multiple healthcare systems. Additionally, while the next phase will involve a pilot project using pseudonymized patient data from a regional hospital, the current study lacks data on how real-time operational demands may affect ITC providers' decision-making. Another limitation is that, based on the complexity of each patient's health conditions, the data extracted from the sending hospital's EHR may vary, necessitating adherence to interfacility transport care protocols.

Conclusion and future directions

The ITC-InfoChain prototype demonstrates the potential of a well-designed architecture based on permissioned blockchain technology to securely manage and share patient data during interfacility transport, particularly for critical care cases. In a simulated environment, the platform successfully met key performance targets, including rapid data recording and retrieval times and strong data privacy protection, achieved by isolating data collection from direct integration with EHR systems. Stakeholder support, particularly from leaders in the critical care units of regional hospital systems, highlights ITC-InfoChain's value in enhancing data accessibility and coordination during emergency transport.

Nevertheless, certain challenges and limitations underscore the need for further development. One critical limitation involves the variability of data extracted from the sending hospital's EHR, as this depends on each patient's specific health conditions. To address this, future implementations must align with Interfacility Transport Care (ITC) protocols, which specify the required types of data based on patient needs, ensuring that shared data is relevant and clinically appropriate. Furthermore, accurately assessing communication and computational costs requires a long-term operational environment, which is outside of the scope of this study.

A proposal for the pilot is in progress, with pending funding to support real-world testing. This pilot will also involve a broader range of stakeholders and allow for detailed cost analysis and assessment of how permissioned blockchain technology can support ITC providers in decision-making, particularly in matching transport resources to patient needs. Targeted education and training for stakeholders, with a focus on pseudonymization features and compliance with privacy standards, are key components of this future phase to build stakeholder confidence and adoption.

Looking forward, ITC-InfoChain offers a scalable, adaptable solution with the potential to extend beyond individual hospital networks, eventually integrating with other healthcare systems to establish a regional or even national framework for interfacility patient transport. By leveraging the flexibility of AWS infrastructure and fostering continuous stakeholder engagement, ITC-InfoChain could serve as a foundational component of secure, interoperable healthcare data systems, paving the way for broader blockchain adoption in healthcare.

Item 20: General (NM)

If applicable, include statements on regulatory approvals (eg, as appropriate, ethical approval, governance approval), trial or study registration (availability of protocol), and conflicts of interest. For implementation reports with a research component, ethical approval or a waiver from an appropriate ethics committee is required. For those without a research component, ethical considerations may still be relevant but do not necessarily require approval or a waiver. Authors may consult Eccles et al [41] for further guidance on ethical considerations in their specific context.

Examples

- "We conducted a randomized controlled pilot trial to assess the SELMA intervention. The clinical trial was approved by the Cantonal Ethics Committee of Zurich (KEK-ZH study protocol identifier Nr. 2017-02136) and was registered at the Swiss National Clinical Trial Portal (SNCTP000002712) and the WHO-accredited German Clinical Trials Register (DRKS00017147). Data protection requirements were fulfilled according to the KEK-ZH" [27].
- "A randomized control trial assessing the effectiveness of the Concerto mobile application on a patient situation awareness score has been designed and should be conducted soon. It will allow for a better evaluation of the cost-effectiveness of such project. Overall, data on the effectiveness of eHealth projects are often lacking, and the creation of the JMIR implementation reports is aiming to fill that gap" [16].
