# Supplementary material for: Appropriately Matching Transport Care Units to Patients in Interhospital Transport Care: Implementation Study
Source: JMIR Form Res. 2024 Dec 13;8:e65626. doi: 10.2196/65626 (PMC11681279; doi:10.2196/65626)
Supplement: Multimedia Appendix 3 [file formative_v8i1e65626_app3.docx]

**This document presents tables detailing the performance measures of the ITC-InfoChain system, highlighting transaction processing times and system adaptability under different configuration settings**.

These findings are supported by images captured during data retrieval and recording while the network, client program and patienTrack app was running on AWS cloud infrastructure (**Appendix C, available upon request).**

**Tables – Column headers:**

**# of TXs submitted**: the number of transactions (TXs) that the client program retrieves from the csv file. **Total time taken for [#] records**: the time it takes the TXs and patient data recorded on the ledger (blockchain, state DB) **Dlt_BatchSiz** (Batch Size in client program’s settings): the number of TXs the client program can process at a time. If there are 50 patients for transport across the netwotk among hospitals, the client program can submit 50 TXs to the network at a time to be recorded in the ledger. **Batch:** a collection of transactions grouped together temporarily to be processed efficiently as a unit **BatchTimout** (second/s): a network setting which determines how long the system waits to fill a batch before sending it, even if the batch size is not met. With a higher BatchSize, the system has more time to collect messages to reach the target size.

The **batching settings** such as **BatchSize (MaxMessageCount, AbsoluteMaxBytes, and PreferredMaxBytes)** areconfigurations managed by the **ordering service** (or "orderer") in Hyperledger Fabric. These parameters determine how the orderer groups TXS into batches before finalizing them into blocks, which are then distributed to the peer nodes and recorded on the blockchain.

**MaxMessageCount**: the **maximum number of TXs** that can be included in a single batch. Larger MaxMessageCount values allow more transactions to be grouped together, which can be beneficial for throughput, especially in high-traffic scenarios.

**AbsoluteMaxBytes**: the **maximum total size in bytes** that a batch can reach. If the combined size of transactions in a batch reaches this limit, the orderer will stop adding more transactions to the batch and will immediately create a block. This parameter ensures that blocks do not exceed a certain size, which is important for preventing oversized blocks that could cause performance and network issues.

**PreferredMaxBytes**: the **preferred size for a batch** in bytes so when the batch reaches this size, the orderer can decide to create a block, even if MaxMessageCount has not been reached. PreferredMaxBytes allows for more flexible batching, especially in scenarios where many small transactions are being processed, optimizing block formation without strictly waiting for the max message count.

| **#** of Transactions  Submitted | Total time taken for [**#**] records  On the ledger | (Dlt_BatchSiz), **#** of TXs the client prog. processes simultaneously | BatchTimeout  (second) | MaxMessageCount  (Block size) | AbsoluteMaxBytes  MB | PreferredMaxBytes  KB |
| --- | --- | --- | --- | --- | --- | --- |
| 1 | 3.19 | 50 | 1 | 20 | 10 | 256 |
| 5 | 3.11 | 50 | 1 | 20 | 10 | 256 |
| 20 | 3.14 | 50 | 1 | 20 | 10 | 256 |
| 100 | 6.36 | 50 | 1 | 20 | 10 | 256 |
| 400 | 24.45 | 50 | 1 | 20 | 10 | 256 |

**Table S1**: **Transaction Processing Times for Various Batch Sizes with Max Message Count of 20 TXs Processed by Client program Simultaneously**

In Table S1, a transaction batch size of 50, with a maximum message count of 20 and a batch timeout of 1 second, resulted in stable performance for lower transaction volumes, achieving approximately 3 seconds for 1 to 20 transactions and around 6 seconds for 100 transactions.

| # of Transactions  Submitted | Total time taken for [#] records  On the ledger | Dlt_BatchSiz , **#** of TXs the client prog. processes simultaneously | BatchTimeout  (second) | MaxMessageCount  (Block size) | AbsoluteMaxBytes  MB | PreferredMaxBytes  MB |
| --- | --- | --- | --- | --- | --- | --- |
| 1 | 3.11 | 50 | 1 | 50 | 20 | 1 |
| 5 | 3.10 | 50 | 1 | 50 | 20 | 1 |
| 20 | 3.13 | 50 | 1 | 50 | 20 | 1 |
| 100 | 6.36 | 50 | 1 | 50 | 20 | 1 |
| 400 | 25.40 | 50 | 1 | 50 | 20 | 1 |

**Table S2** explored a similar configuration but doubled the maximum message count to 50, slightly reducing latency and enhancing throughput efficiency

In Table S2, a transaction batch size of 50, with a maximum message count of 50 and a batch timeout of 1 second, resulted in stable performance for lower transaction volumes, achieving approximately 3.1 seconds for 1 to 20 transactions and around 6.36 seconds for 100 transactions. For higher transaction volumes, such as 400 transactions, the processing time increased to approximately 25.40 seconds, indicating that while the increased message count improved throughput at moderate levels, its impact was less pronounced at higher transaction volumes without further configuration adjustments.

| # of Transactions  Submitted | Total time taken for [#] records  On the ledger | (Dlt_BatchSiz), **#** of TXs the client prog. processes simultaneously | BatchTimeout  (second) | MaxMessageCount  (Block size) | AbsoluteMaxBytes  MB | PreferredMaxBytes  MB |
| --- | --- | --- | --- | --- | --- | --- |
| 1 | 3.11 | 100 | 1 | 50 | 20 | 1 |
| 5 | 3.11 | 100 | 1 | 50 | 20 | 1 |
| 20 | 3.12 | 100 | 1 | 50 | 20 | 1 |
| 100 | 3.29 | 100 | 1 | 50 | 20 | 1 |
| 400 | 13.12 | 100 | 1 | 50 | 20 | 1 |

**Table S3: Optimized Performance Results with Batch Size of 400 and Max Message Count of 100 TXs Processed by Client Simultaneously, Highlighting Reduced Latency at Higher Transaction Volumes**

In Table S3, a significantly larger transaction batch size of 400 and maximum message count of 100 demonstrated marked improvements in scalability, achieving processing times under 2 seconds for 400 transactions.

| # Transactions  Submitted | Total time taken for [#] records  On the ledger | (Dlt_BatchSiz), **#** of TXs the client prog. processes simultaneously | Dlt_BatchSiz Settings.cfg | MaxMessageCount  (block size) | AbsoluteMaxBytes  MB | PreferredMaxBytes  MB |
| --- | --- | --- | --- | --- | --- | --- |
| 1 | 3.10 | 400 | 1 | 100 | 20 | 1 |
| 5 | 3.09 | 400 | 1 | 100 | 20 | 1 |
| 20 | 3.11 | 400 | 1 | 100 | 20 | 1 |
| 100 | 3.29 | 400 | 1 | 100 | 20 | 1 |
| 400 | 1.8 | 400 | 1 | 100 | 20 | 1 |

**Table S4: System Performance with Batch Size of 400 and Max Message Count of 200 TXs Processed by Client Simultaneously, Demonstrating Consistent Low Latency for Large Transaction Batches**

Table S4 further increased the maximum message count to 200, maintaining sub-2-second performance for high transaction loads, highlighting the system's ability to handle extensive data input with minimal latency.

| # Transactions  Submitted | Total time taken for [#] records  On the ledger | (Dlt_BatchSiz), **#** of TXs the client prog. processes simultaneously | Latency  (second) | MaxMessageCount  (block size) | AbsoluteMaxBytes  MB | PreferredMaxBytes  MB |
| --- | --- | --- | --- | --- | --- | --- |
| 1 | 3.10 | 400 | 1 | 200 | 20 | 1 |
| 5 | 3.10 | 400 | 1 | 200 | 20 | 1 |
| 20 | 3.12 | 400 | 1 | 200 | 20 | 1 |
| 100 | 3.29 | 400 | 1 | 200 | 20 | 1 |
| 400 | 1.9 | 400 | 1 | 200 | 20 | 1 |

**Table S5: Comparison of Transaction Processing Times Across Different Configurations, Emphasizing High Throughput Achieved with Larger Batch and Message Count Settings**

Table S5 confirmed that optimized configurations using larger batch and message counts consistently achieve the best performance, reinforcing the importance of these parameters in tuning Hyperledger Fabric for high-throughput applications. These results indicate that the system's configuration flexibility allows it to scale efficiently, making it well-suited for applications requiring reliable and rapid transaction processing.

Data Analysis: The prototype demonstrated exceptional scalability and efficiency. Key performance benchmarks were achieved, with processing times consistently averaging around 3 seconds for smaller transaction volumes (1, 5, and 20 transactions) and approximately 6 seconds for moderate volumes (100 transactions) under an initial configuration using a batch size of 50. When the system was further optimized with a batch size of 400 and a higher transaction message count limit, it showcased impressive scalability, processing 400 transactions within a remarkably low 1.8 to 1.9 seconds. This optimization reveals the configuration’s capability to handle extensive transaction loads while maintaining efficiency, making it a powerful solution for high-volume, time-sensitive applications.
